# Generating STEC-Specific *Ackermannviridae* Bacteriophages Through Tailspike Protein Chimerization

**DOI:** 10.3390/v17121614

**Published:** 2025-12-14

**Authors:** Jose Gil, John Paulson, Henriett Zahn, Matthew Brown, Minh M. Nguyen, Stephen Erickson

**Affiliations:** 1 Labcorp, Calabasas, CA 91301, USA; gilj@labcorp.com; 2 Labcorp, New Brighton, MN 55112, USA; paulsoj@labcorp.com (J.P.); zahnh@labcorp.com (H.Z.); nguyem5@labcorp.com (M.M.N.); 3 Labcorp, Burlington, NC 27215, USA; matthewjudebrown@gmail.com

**Keywords:** phage-based detection, bacteriophage, luciferase reporter phage, *Ackermannviridae*, receptor-binding protein, tailspike protein, *Escherichia coli*, *Kuttervirus*, STEC, CBA120, TSP

## Abstract

Shiga toxin-producing *Escherichia coli* (STEC) pose a significant threat to public health and effective methods of detection are needed. The use of naturally occurring bacteriophages (phages) to detect *E. coli* has been well documented. However, detecting multiple serotypes at the same time often required multiple phages specific to individual serotypes. To limit the burden of complex cocktails, this study aimed to engineer phages with an expanded host range that allows each phage to contribute to detection across multiple STEC serogroups. *Kutterviruses*, in the *Ackermannviridae* family, contain four tailspike proteins (TSPs), each of which confers tropism to a different bacterial strain. The modular nature of TSPs allows for mixing receptor-binding domains from diverse phage types. The host range of the *Kuttervirus* CBA120 was modified by replacing its native tailspike proteins (TSPs) with chimeric versions incorporating receptor-binding domains from related and unrelated phages. A structure-guided approach was utilized to overcome minimal sequence similarity between donor and recipient phages and achieve novel functional TSP chimeras. Two engineered phage variants were created that collectively detect five STEC serogroups: O26, O45, O103, O111, and O157. Spotting and luciferase assays confirmed that the replacement TSPs were functional and the phages had acquired new host ranges. This study demonstrates the feasibility of engineering *Ackermannviridae* phages with customized host ranges for detecting multiple STEC strains. This approach has potential applications in developing improved phage-based bacterial detection, therapy, and biocontrol.

## 1. Introduction

Shiga toxin-producing *Escherichia coli* (STEC) are a group of foodborne pathogens that pose a significant threat to public health. *Escherichia coli* (*E. coli)* O157 and the “Big Six” non-O157 STEC serogroups (O26, O45, O103, O111, O121, and O145) account for the majority of STEC infections in humans, causing severe illnesses such as hemorrhagic colitis and hemolytic uremic syndrome [1]. These pathogens are responsible for numerous foodborne outbreaks, resulting in substantial economic losses and public health concerns [2,3]. The detection and control of STECs in the food supply chain is therefore of paramount importance for ensuring food safety and protecting consumer health.

Bacteriophages (phages) have emerged as powerful tools with significant potential within the commercial food industry, with uses in such areas as biocontrol [4] and detection of foodborne pathogens [5,6,7,8]. Phage technology has also been applied to bioremediation [5,9], and has a long history and recent resurgence for clinical phage therapy [10,11,12,13]. In particular, phage-based detection systems [7,8,14,15] have gained renewed interest due to recent advancements in reporter technologies, such as the engineered NanoLuc^®^ luciferase [16], which offers unprecedented sensitivity and a small genetic footprint.

The performance of phage-based technologies is intrinsically linked to their host specificity, which is largely determined by receptor-binding proteins (RBPs) such as tailspike proteins (TSPs) [17,18]. TSPs are structurally conserved proteins that form homotrimers, consisting of an N-terminal “head” domain that binds to the phage baseplate and a C-terminal β-barrel domain responsible for receptor binding and enzymatic activity [18]. The modular nature of TSPs has made them attractive targets for genetic manipulation to modify phage host ranges [14].

While many phage engineering efforts focus on modifying a single RBP [19,20,21], some phages, specifically *Kutterviruses* in the *Ackermannviridae* family [22,23], encode multiple TSPs (Figure S1) that can recognize serologically distinct LPS oligosaccharides (O-antigens) [24] as host receptors. The well-characterized *Kuttervirus* CBA120 [23] encodes four TSPs (TSP1-4) that collectively enable infection of *Salmonella enterica* serovar Minnesota and multiple *Escherichia coli* serovars (O157, O77, and O78) [25]. The native multi-host capacity of CBA120 implicates the *Ackermannviridae* family as a promising platform for engineering synthetic phages with custom-tailored host ranges. This characteristic offers unique advantages for developing versatile detection and biocontrol agents.

Though the Ackermannviridae family is well characterized, with over two hundred genomes publicly described on Genbank [26], we were unable to find references for TSPs against all of the Big Six STEC O-antigens within the family. Specifically, genome descriptions did not mention tropism to O26, O45, O103, nor O121, nor were they found in the literature [22]. Thus it was necessary to expand the search to unrelated phages, such as podoviruses, where matches were found [27].

In this study, our goal was to adjust the native host range of CBA120 to eliminate host recognition of *Salmonella* and non-STEC *E. coli,* while maintaining and expanding coverage of the dominant STEC-specific serogroups. We applied TSP engineering techniques previously used to generate *Salmonella* specific *Kuttervirus* SPTD1 [8,14], to incorporate TSPs from *Ackermannviridae* and non-*Ackermannviridae* phages to generate functional chimeric proteins. We demonstrate that by replacing native TSPs targeting non-STEC species with chimeric TSPs built from the receptor-binding domains of diverse STEC-specific phages, we can successfully shift the host range of CBA120 to STEC-specificity. Importantly, we show that with the use of two engineered CBA120 variants, combined with the sensitivity of the NanoLuc^®^ reporter system, we can collectively detect five of the target STEC serogroups (O26, O45, O103, O111, and O157). This expanded detection range can provide a rapid screening method for STECs in food samples. Our findings highlight the potential of the *Ackermannviridae* family as a versatile platform for generating synthetic phages with tailored host ranges. By leveraging the modular nature of TSPs across phage families and the multi-TSP architecture *of Ackermannviridae* phages, we demonstrate a novel approach to phage engineering that could have broad applications in food safety, diagnostics, and biocontrol.

## 2. Materials and Methods

### 2.1. Bacterial Strains

Source and strain information for bacterial species utilized in this study is provided separately (Table S1). Bacterial cultures were grown at 37 °C in tryptic soy broth (TSB) (Oxoid, Hampshire, UK) for day growth or slow-growing strains or in GIBCO Luria-Bertani (LB) broth for overnight cultures (Thermo Fisher Scientific, Waltham, MA, USA), with shaking at 225 revolutions per minute (RPMs).

### 2.2. DNA, Amino Acid, and Structural In Silico Comparisons

The genomes of bacteriophages CBA120 (NC_016570) [23], STP55 (OM688977.1) [28], Ro45lw (MK301532.1) [29,30], Ro103C3Iw (MN067430.1) [31], and the bacterial strain *E. coli* O26 RM10386 (NZ_CP028126.1) are publicly available on NCBI’s GenBank [26]. Sequence comparisons between the TSPs were performed using EMBOSS Needle Pairwise Alignment [32,33]. Determination of N- and C-terminal domains were based upon known crystal structures of CBA120’s TSP1-4 [25,34,35,36] or protein structural predictions generated with AlphaFold2 for the remaining phage TSPs and proposed chimeric TSPs [37]. Alphafold2 was run on Ubuntu Linux 22.04 in multimer mode with the reduced database (DBS). Structures were compared using Pairwise Structure Alignment [38]. Output files were then visualized within Mol* 3D Viewer on the RCSB website [39]. Sequence manipulation was performed with A Plasmid Editor [40] and visualized with SnapGene Viewer (SnapGene, Boston, MA, USA). Adobe Photoshop (Adobe, San Jose, CA, USA) and PowerPoint 2019 (Microsoft, Redmond WA, USA) were used to finalize figure preparation as needed.

### 2.3. Phage Enumeration

Bacteriophages used in this study were enumerated using three techniques. Plaque forming unit (PFU) concentration was determined by standard double-layer agar plaque assays on either TSB or LB agar plates with 3ml molten 0.5% agar TSB or LB (BD Difco, Franklin Lakes, NJ, USA) using the appropriate bacterial strain to seed the lawns. Two methods based on the Tissue Culture Infectious Dose 50 (TCID_50_) assay were also utilized, and the concentration determined using the Spearman–Karber method [41,42]. Briefly, the TCID_50_ assay is a limiting dilution method where a virus stock is serially diluted then aliquoted to wells on a 96-well plate followed by addition of host cell culture. Any wells that show clearance or lysis after an overnight incubation are scored as infected, and the ratio of infected and uninfected wells are used to back calculate the 50% probability of infection per unit volume (TCID_50_/mL).

The first TCID_50_-based method was used to determine the concentration of transducing units based on expression of the luciferase reporter gene, termed Transducing Unit 50 per mL (TU_50_/mL). 100 µL per well of phage in 1:10 serial dilution from 10^−1^ to 10^−8^ were aliquoted by row into a 96-well luminometer plate, in six replicate columns. Host bacteria were grown in LB at 37 °C with 225 rpm shaking until visibly turbid, then diluted 1:10 in LB. 50 µL of the diluted culture was seeded to each well in 5 columns, with the sixth column receiving LB only as a negative control. After an overnight room temperature incubation, Promega NanoGlo^®^ assays were performed with 25 µL of 1× Nano-Glo^®^ substrate (Promega Corp., Madison, WI, USA) in either a Promega GloMax 96 or Promega GloMax Navigator luminometer (Promega Corp, Madison, WI, USA).

The second TCID_50_-based method that was used to determine the concentration of recombinant phage infectious units was based on PCR detection of the recombined TSP gene, and will be subsequently referred to as PCR Infectious Unit 50 per mL (PCRIU_50_/mL). After completion of the TU_50_ assay described above, 5 µL of each well was diluted 1:10 in molecular grade water, and used as a template for PCR using FastStart Taq (Roche, Basel, Switzerland) and the indicated primers (Table S2) with standard FastStart Taq conditions with a 55 °C annealing temperature and up to 45 cycles. Samples were loaded on 1% to 1.5% agarose gels in 1.0× TAE (Tris-acetate-EDTA) with SybrSafe DNA stain (Thermo Fisher Scientific, Waltham, MA, USA), 48 wells at a time. DNA bands at the expected amplicon size were scored as positive infectious units, and the ratio of positives to negatives was used to back calculate the initial concentration of PCRIU_50_/mL using the Spearman–Karber method. Background amplification from the no-cell control column was used to delineate a threshold of detection for viable recombinant phage, as unpackaged recombined genomes can serve as a template for PCR, but are not replicated during the infection to higher concentrations.

### 2.4. Phage Engineering

The *E. coli* phage CBA120 was obtained from Dr. Elizabeth Kutter [23]. The parental phage used was CBA120.NL, which is the CBA120 phage with the Nanoluc^®^ reporter placed downstream of the major capsid protein, and which has been sequence verified [8,14]. The same methods were used as previously described, with the use of an expanded set of bacterial strains [14,23]. *E. coli* O157, *E. coli* O45, *E. coli* O111, *E. coli* O103, and *E. coli* O26 strains from the American Type Culture Collection (ATCC, Manassas, VA, USA) were used for plasmid transformation and homologous recombination infection and subsequent recombinant purification and screening. During the phage engineering phase, bacteria were grown in LB broth with 100 µg/mL carbenicillin for plasmid maintenance. All plasmids used to generate the chimeric phage were synthesized by GeneWiz (GeneWiz, South Plainfield, NJ, USA) using their PriorityGENE service, with custom designed inserts into their pUCGW-Amp plasmid, and verified by Sanger sequencing by the manufacturer (Table S2). The sequence of pUCGW-Amp is publicly available from GeneWiz.

#### 2.4.1. O45-specific RBP-CBA120-3 Construction

RBP-CBA120-3 used CBA120.NL as the parental phage with a chimeric TSP1, consisting of an N-terminal region from CBA120’s TSP1 and a TSP C-terminal region from the podovirus *Escherichia* phage vB_EcoP-Ro45lw (Ro45lw) [29,30] gp44 (Table S2). The transition site between CBA120 and Ro45lw TSP was placed after Leu163 at the end of a predicted alpha-helix in the “neck” or hinge region based on a Pairwise Structure Alignment [38] of the published crystal structure of CBA120 TSP1 [34] and an AlphaFold2 [37] prediction of the Ro45lw TSP trimer, separating the N- and C-terminal functions [25,35].

To generate the recombinant phage, homologous recombination was facilitated by generating a plasmid with the Ro45lw TSP C-terminal sequence flanked with 450 bp of identical sequence upstream and downstream of the wild-type CBA120 TSP C-terminal end (Figure S2). The upstream homologous region does not include the ATG start site to ensure plasmid-based expression of the chimeric TSP does not provide the full-size protein in trans to the parental phage, requiring recombination to generate the chimeric TSP. This approach has been described previously in detail [8]. *E. coli* O157 strain ATCC 43888 was transformed with the plasmid via electroporation using a Bio-Rad GenePulser (Bio-Rad Laboratories, Hercules, CA, USA) electroporator as described previously [8,14,15]. Briefly, RBP-CBA120-3 was generated via infection of the transformed cells grown in TSB with 100 µg/mL carbenicillin, with CBA120.NL, leading to homologous recombination between the infecting phage and plasmid, then recombinants were isolated by plaque assay of filtered lysate on *E. coli* O45 ATCC BAA-2193 host cells. Plaques were picked and sequentially passaged at least three times for clonal purity as previously described [8,14].

#### 2.4.2. O111-specific RBP-CBA120-5 Construction

RBP-CBA120-5 was generated with the following differences. The chimeric TSP4 consisted of an N-terminal region from CBA120’s TSP4 (ORF213) and a C-terminal region from closely related *Kuttervirus STP55’s* TSP4 [28] (Table S2, Figure S3). A similar plasmid was designed comprising the sequence encoding for the C-terminal region of SPT55’s TSP4 (nt 1471-3036) flanked between 500 bp upstream and the downstream matching sequences. *E. coli* O157:H7 strain ATCC 43888 was transformed with the STP55 plasmid and infected with RBP-CBA120-3. Lysates were plated and plaques selected on *E. coli* O111 (ATCC BAA-2201). DNA from purified lysate was sequence verified using the Illumina MiSeq protocol by Laragen (Laragen Inc., Culver City, CA, USA).

#### 2.4.3. O103-specific RBP-CBA120-6 Construction

To generate RBP-CBA120-6, Pairwise Structure Alignment was again performed with Ro103C3Iw TSP and all four CBA120 TSPs to select the best structural match for a chimera. The N-terminal region from CBA120’s TSP3 and a C-terminal region from the TSP from *Kayfunavirus Escherichia* phage vB_EcoP-Ro103C3Iw (Ro103C3Iw) (Table S2, Figure S4) were used to generate the chimeric TSP. The same homologous recombination infection method as previously described was used, with the following differences. Recombination was performed in *E. coli* O45 BAA-2193 bacteria, as it was found it generated larger plaques and better yield compared to the original host. Lysates were plated on *E. coli* O103 strains ATCC BAA-2210 and BAA-2214, to select for recombinants, but yielded no plaques. PCR primers were designed to detect the recombinant TSP (Table S3) instead, and in parallel with TU_50_ assays, PCRIU_50_ were performed to determine the ratio of TU_50_ to PCRIU_50_ and verify recombination.

Limiting Dilution Enrichments (LDEs) were performed similarly to the method previously described [8,14], but were based on the presence of a PCR product rather than luciferase activity. Briefly, based on the detected concentration of PCRIU_50_, 96-well plates were prepared with multiple dilutions of the lysate in LB with *E. coli* O45 BAA-2193. As previously reported, overnight incubation at room temperature was sufficient to detect luciferase far over background in positive wells [8,14], so the same conditions were used for the PCR method. Lysate samples from each plate well were diluted 1:10 in water, then subjected to PCR to detect the presence of recombinants as described in the PCRIU_50_ assay. Remaining lysate from multiple positive wells from the higher dilution plate, which would have fewer parental input phages, were harvested separately, diluted to 500 µL in TMS (50 mM Tris-HCl pH 7.8, 10 mM MgCl2, and 300 mM NaCl), centrifuged at 6800× *g* for 2 min, and filtered through a Millipore 0.45 µm Ultra-Free MC spin filter (MilliporeSigma, Burlington, NC, USA).

The filtered lysates were then subjected to a TU_50_ and PCRIU_50_ assays to determine the total phage to recombinant phage ratio for each sample. The sample with the highest recombinant ratio was used in the next enrichment round LDE. This was repeated until the ratio dropped enough to screen individual plaques. Forty-six plaque stabs and a negative control agar stab were suspended in 50 µL of TMS each. Five microliters of the phage suspension were added to 48 wells in luminometer plates with either *E. coli* O45, as a positive control, or O77, to assess for loss of native TSP3 tropism, with a 48th well spiked with parental phage as a positive control (Figure S5). After an overnight room temperature incubation, 25 µL of 1× Nano-Glo^®^ substrate (Promega Corp., Madison, WI, USA) was added to each well and luciferase activity was measured using either a Promega GloMax 96 or Promega GloMax Navigator luminometer. The ratio of O45 to O77 luciferase activity was calculated and wells with lower relative O77 luciferase activity were harvested as above as candidate recombinant isolates. Five microliter samples were used in a three-hour 37 °C infection against O45 and O77 to verify loss of O77 luciferase activity. Lysates from these infections were diluted in water and subjected to PCR as described above to verify recombinants.

Three rounds of plaque assay passages were performed and the loss of luciferase activity in O77 bacterial strain samples was used as verification of recombinant clonal purity, with the final plaque suspended in TMS and filtered as described above. Broth lysate was prepared by infecting 1 mL of *E. coli* O45 in LB diluted 1:100 from an overnight culture with 5 µL of the final plaque suspension for three hours at 37 °C with 225 rpm shaking, centrifuged for two minutes at 6800 g, and filtered at 0.45 µm. To verify gain of function of the recombinant to infect *E. coli* O103, 5 µL of the broth lysate was used to infect a 1 mL 3 h 37 °C culture of *E. coli* O103 BAA-2210 in a 2 h time course infection (Figure S6).

#### 2.4.4. O26-specific RBP-CBA120-9 Construction

RBP-CBA120-9 was generated using the N-terminal region from CBA120’s TSP3 and a C-terminal region from a prophage TSP in *Escherichia coli* strain RM10386 (Table S2, Figure S7). Homologous recombination was performed as described previously using RBP-CBA120-5 as the parental phage. Lysates were plated on *E. coli* O26 strains BAA-2205, BAA-2212, BAA-2196, and May063 to select for recombinants. As with the generation of RBP-CBA120-6, no plaques were observed, so the PCR-based method was again used to isolate the recombinant. A 1 mL broth lysate was prepared as described earlier, and the NanoLuc^®^ background was reduced via buffer exchange with TMS using 100 kD molecular weight cutoff Pierce Protein Concentrators (Thermo Fisher Scientific, Waltham, MA, USA) as per user manual with 3 × 4 mL TMS washes, and tested against *E. coli* O26 BAA-2205 and *E. coli* O77 to verify expected activity of the recombinant (Figure S8).

### 2.5. Phage Stock Preparation

High titer broth lysates were generated and purified as previously described [15] for all phages used in this study with the exception of RBP-CBA120-3 (which did not undergo purification past broth lysate preparation). Lysates were prepared with different *E. coli* host strains as needed. CBA120.NL, RBP-CBA120-6, and RBP-CBA120-9 were prepared in *E. coli* O157 43888, RBP-CBA120-3 was prepared in *E. coli* O45 (BAA-2193), and RBP-CBA120-5 was prepared in *E. coli* O111 (BAA-2201).

### 2.6. Phage Spot Assay

Bacterial susceptibility to phage infection was evaluated using a double-layer agar method. Bacterial cultures in log growth phase were diluted to an OD_600_ of 0.2 in tryptic soy broth (TSB). A semi-solid layer was created by mixing 300 µL of bacterial suspension with 3 mL of molten 0.5% weight/volume TSB agar (BD Difco, Franklin Lakes, NJ, USA), which was then poured over a solid TSB agar base. A total of 4 µL of the phage diluted to 1 × 10^8^ PFU/mL was applied to designated areas on the semi-solid layer. Plates were incubated overnight at 37 °C and were imaged using a Gel Doc EZ Imager (Bio-Rad Laboratories, Hercules, CA, USA).

### 2.7. Luciferase Reporter Phage Assays

Tropism of purified phage stocks was verified by luciferase assay [14]. Working stocks at 1.2 × 10^7^ PFU/mL were prepared as previously described [15]. Log phase cultures of target bacteria were diluted to 0.2 OD_600_, then 100 µL aliquots were transferred into 96-well luminometer plates and infected with 10 µL working phage stocks, incubated at 37 °C for 2 h, and assayed for luciferase activity as previously described [14].

## 3. Results

### 3.1. Selection of Bacteriophage C-Termini for Chimerization

CBA120 was identified as an ideal foundation for engineering a multi-host STEC-specific phage reporter. The O-antigen targets of each TSP in wild-type CBA120 are *Salmonella enterica* Minnesota O:21 for TSP1 [14,25], *E. coli* O157 for TSP2 [25], *E. coli* O77 for TSP3, and *E. coli* O78 for TSP4 [25]. For the purpose of this study, only *E. coli* O157 tropism was desirable as this serogroup is associated with STEC and is an important food pathogen. To identify TSP donors for engineering, a literature search for other STEC-specific phages with TSPs was performed. Candidates were found for an additional four STEC serogroups. A TSP for O26 was found in a *Lederbervirus* prophage within *E. coli* strain RM10386, a TSP for O45 was found on the *Kayfunavirus* phage Ro45lw, a TSP for O103 was found on the *Kayfunavirus* phage Ro103C3Iw, and a TSP for O111 was found on the *Kuttervirus* STP55 [27,28,29,31]. Table 1 lists the source of chimeric TSPs for each recombinant bacteriophage in the study.

### 3.2. Structural Comparison of CBA120’s Tailspike Proteins to Unrelated Phage

**Figure 1:**
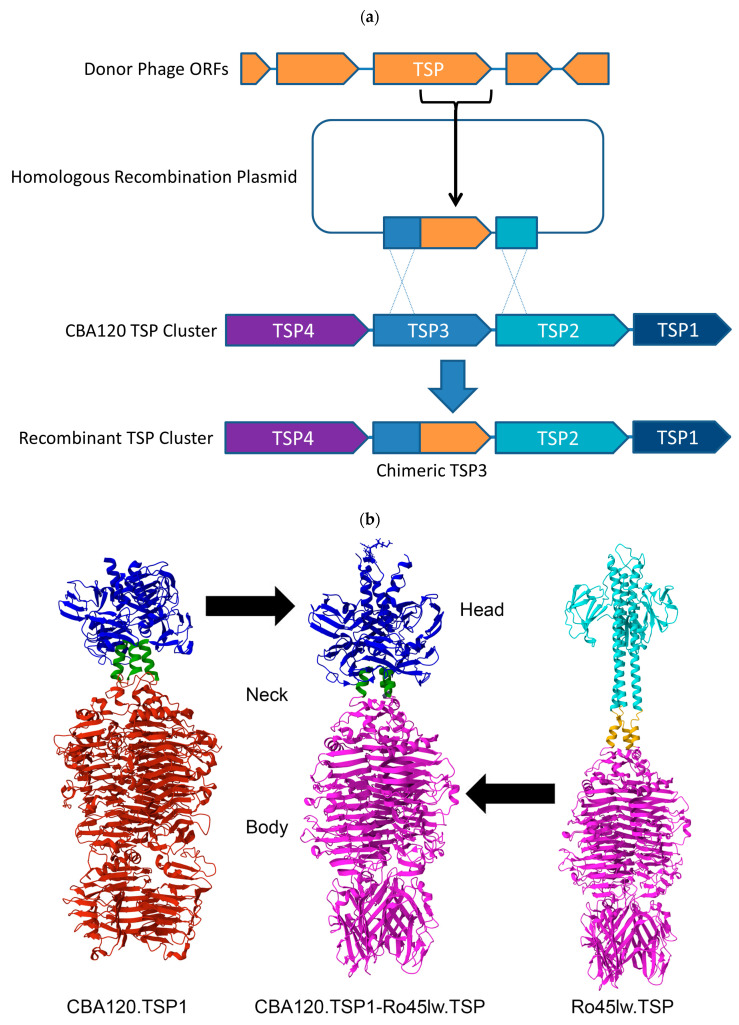

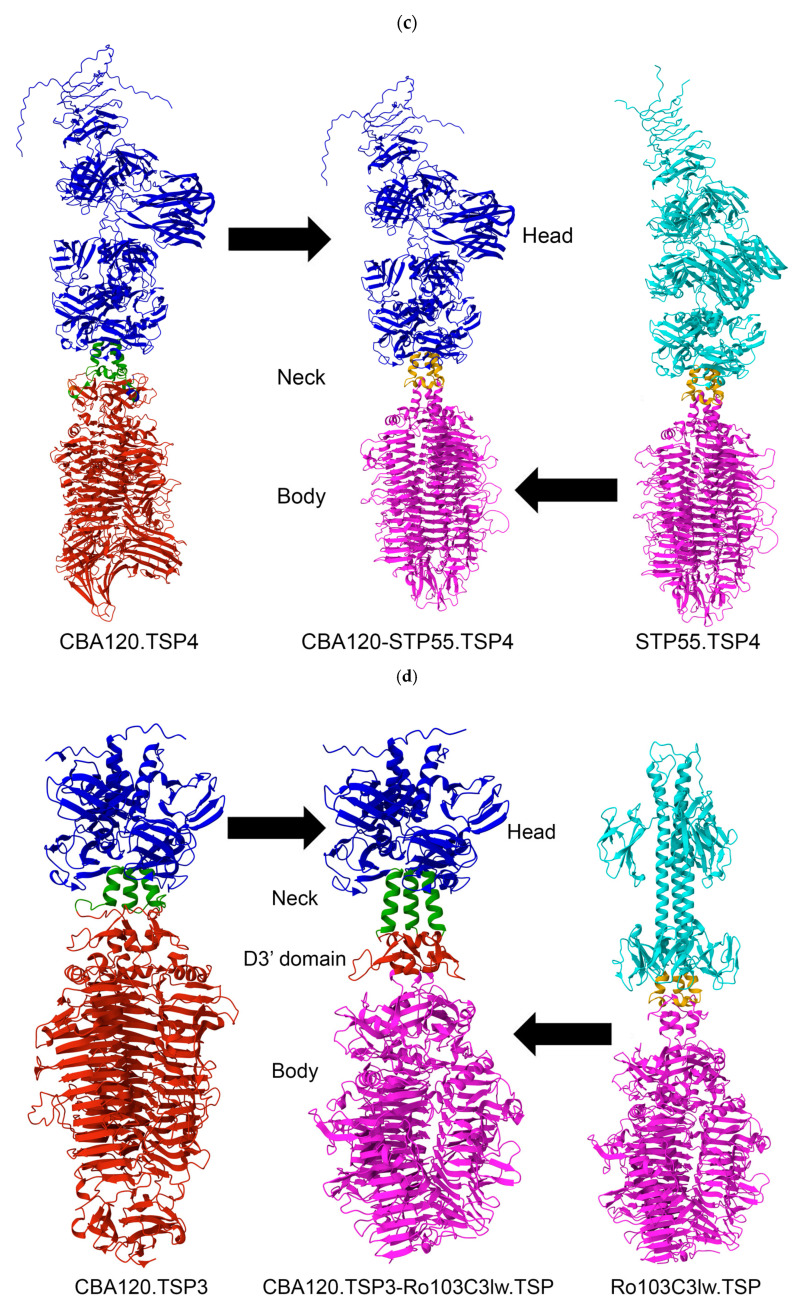

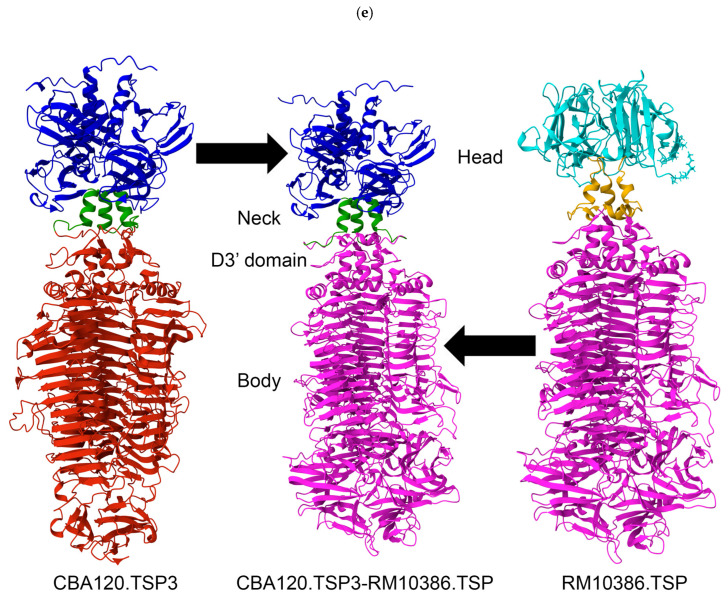
Chimeric TSPs are generated via homologous recombination using the target TSP’s N-terminal region and downstream ORF’s N-terminal region for cross-over regions, with TSP3 shown as an example (**a**) with an unrelated phage. (**b**) Generation of the chimeric O45-specific TSP1 incorporated the attachment domain (head) and neck of CBA120.TSP1 (ORF210) and catalytic domain (body) of Ro45lw TSP (gp44). (**c**) O111-specific TSP4 composed of the CBA120.TSP4 (ORF213) head domain and STP55 (ORF00054) neck and body domain. (**d**) The O103-specific TSP3 was generated with the head, neck, and D3′ stability domain of CBA120.TSP3 (ORF212) and Ro103C3Iw TSP (Ro103_45) body. (**e**) The O26-specific chimeric TSP3 was generated with a prophage *Lederbergvirus* TSP from *E. coli* O26 strain RM10386 (I3U_RS19900). Crystal structure of CBA120 TSP1 are depicted, all others were generated with AlphaFold2 [37]. CBA120 TSP head, neck, and body domains are depicted in blue, green, and red, respectively. Donor TSP head, neck, and body domains are depicted in cyan, yellow, and magenta, respectively.

Recombination between Kuttervirus TSPs is relatively straightforward due to high sequence identity in the N-terminal attachment domains, and has previously been described [14]. With the exception of *Kuttervirus* STP55, the other TSPs show little to no sequence similarity via pairwise amino acid sequence alignment to any CBA120 TSP (Table 2). Alternatively, structural alignment was performed using AlphaFold2 structural predictions with CBA120 TSP structures (Figure 1). When high structural similarity was found between TSPs, splice borders were then chosen based on structural overlap within the neck region (Figures S2C and S4D) or nearby domain transitions.

### 3.3. Generation of Chimeric Ackermannviridae Tailspike Proteins

Natural horizontal gene transfer between TSPs has been proposed to occur downstream of the conserved N-termini, providing a path to altered host range via swapping C-terminal receptor-binding and catalytic domains [22]. Although previously attempted, artificial engineering between diverse bacteriophage families has encountered difficulties [43]. While an O111-specific TSP can be found in a closely related phage to CBA120, it was necessary to search outside of the *Ackermannviridae* family in order to modify its tropism to target O45, O26, and O103 strains of *E. coli*, as none have been described. An extensive list of TSPs, and their predicted O-antigen targets [27] were researched, yielding candidate TSPs from unrelated podoviruses (*Kayfunavirus* and *Lederbervirus*).

Utilizing unrelated phages as TSP donors introduces significant challenges. For example, the TSP from vB_EcoP-Ro45lw (Ro45lw.TSP) showed no substantial amino acid sequence similarity to the CBA120 TSPs (Table 2), negating the prior method used to determine the splice site to generate the chimeric TSP [14]. As TSP structures are well conserved [22], a structure-based method was employed using AlphaFold2-derived protein structural predictions (Figure 1b–e), allowing for the delineation of the various domains, including the N-terminal attachment (head) domains and C-terminal catalytic and receptor-binding domains [36]. The crystal structure of CBA120’s TSP1 (CBA120.TSP1) was aligned against the predicted structure of Ro45lw.TSP, which demonstrated conserved structural elements, even with little amino acid sequence conservation (Figure S2b). CBA120.NL, a previously described luciferase reporter containing the wild-type TSP operon (Figure S1), served as the parental phage to generate RBP-CBA120-3, substituting *E. coli* O45 specificity for the native *Salmonella* specificity via homologous recombination at TSP1 (Figure S2). As in our previous study [14], recombinants were selected from infection lysate by plaque assay on the new target host, *E. coli* O45 BAA-2193, which demonstrated successful generation of the chimera, due to gaining the donor TSP’s O-antigen specificity.

To confer O111 tropism, RBP-CBA120-5 was generated by modifying TSP4 from RBP-CBA120-3 (Figure S3). The C-terminal catalytic domain of TSP4 from the related *Kuttervirus* STP55 (Figure S1) was used due to the high sequence identity with the CBA120 TSP4 N-terminus (Figure S3B). Successful detection of plaques upon *E. coli* O111 demonstrated generation of a functional recombinant, due to gaining the donor TSP’s O-antigen specificity. The genome of RBP-CBA120-5 was found to match the expected sequence, further demonstrating successful generation of the chimeric protein by our methods.

The O103-specific TSP from Ro103C3Iw was the donor TSP for RBP-CBA120-6, replacing native *E. coli* O77 tropism (Figure S4). To choose which CBA120 TSP to chimerize, a new strategy, leveraging pairwise structure alignment [38] of the O103-specific TSP against the CBA120 TSPs, and picking the best match in the neck region (Figure S4D) was used to select TSP3. The splice location was chosen to incorporate the parental D3’ domain, a likely stability motif [44] (Figure 1d). Surprisingly, selection of the recombinant on *E. coli* O103 strains failed to yield any plaques from the infection lysate. One plausible explanation is that the chimeric TSP is generated but unable to propagate in *E. coli* O103 due to an inability to complete a full infection cycle. This may be a result of an inability to adsorb due to a poorly engineered or poorly chosen chimeric TSP, an inability to complete productive infection due to host immunity, [45] or other natural resistance. To test this hypothesis, PCR was used to successfully detect the chimeric TSP (Figure S4E) with recombinant specific primers (Table S4) in infected bacteria and lysates.

**Figure 2:**
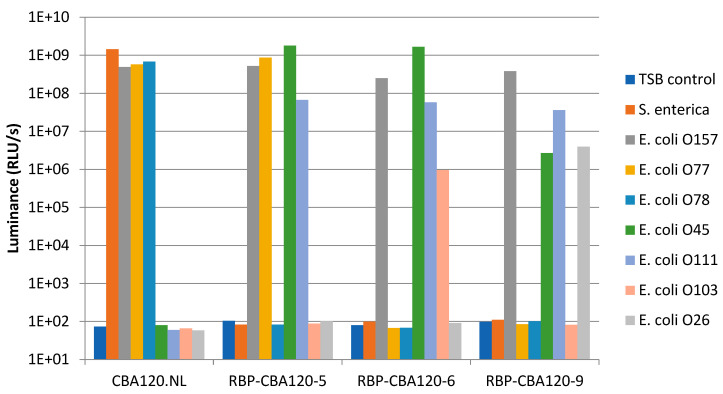
Luciferase activity-based tropism of purified recombinant phage stocks. A total of 1.2 *×* 10^5^ PFU of each phage was added to indicated bacterial strains. Replacement of wild-type TSPs with chimeric TSPs are evident in strain-specific signal profiles for each recombinant phage. Data in table form are provided in Table S5.

Purified recombinant phage was tested against *E. coli* O103 BAA-2210 in a two-hour time course infection (Figure S6), yielding increasing luciferase signal over sixty minutes. The inability of RBP-CBA120-6 to form plaques on *E. coli* O103 but express luciferase indicates that the recombinant is able to adsorb and infect the host but is unable to complete an infection cycle. The lack of luciferase signal from off-target negative control strains (Figure 2) demonstrate a non-specific effect, such as natural competency, is unlikely. Expression of luciferase requires the transfer of the phage genomic DNA into the host bacteria, which is dependent on phage adsorption, and appears to be O-antigen specific.

To complement RBP-CBA120-6 in a possible STEC detection phage cocktail, RBP-CBA120-9, with the O26-specific TSP from a *Lederbergvirus* prophage in *E. coli* O26 strain RM10386, was generated. The same methods were employed to generate the recombinant (Figure S7 and Figure 1e). This also resulted in the absence of plaques on the O26 target strains, requiring PCR-based methods to isolate the recombinant. *E. coli* O26 specificity was verified by detection of luciferase signal after a two-hour infection (Figure S8).

### 3.4. Chimeric Phage Viability on Target Bacterial Strains

**Figure 3:**
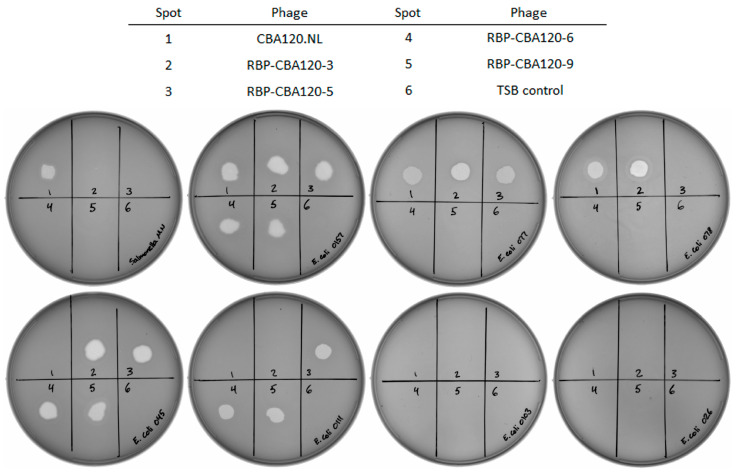
Spot assays on bacterial lawns of each pure recombinant broth lysate indicate the ability of each to form plaques, demonstrating the inability of RBP-CBA120-6 or RBP-CBA120-9 to efficiently lyse their respective targets of *E. coli* O103 or *E. coli* O26. Plates were imaged using a Gel Doc EZ Imager (Bio-Rad Laboratories, Hercules, CA, USA). From left to right, **top**: *Salmonella enterica* Minnesota, *E. coli* O157, *E. coli* O77, and *E. coli* O78. From left to right, **bottom**: *E. coli* O45, *E. coli* O111, *E. coli* O103, and *E. coli* O26. The legend indicates the recombinant by spot number. Bacterial strain information is provided separately (Table S1).

RBP-CBA120-6 and RBP-CBA120-9 did not form plaques from the initial homologous recombination lysate on their new target strains, O103 and O26, respectively. This may be due to poor efficiency of plating and/or too few recombinant virions due to poor recombination efficiency. Alternatively, chimeric phage may not be capable of completing the infection cycle in these strains, possibly due to acquired immunity [45] or other resistance mechanisms. To eliminate concerns regarding efficiency of plating or low concentration, high-titer purified phage stocks were used to test for viability. Bacteriophage spot assays were performed [46] to determine viability of the chimeric bacteriophage. For spotting assays, approximately 4 × 10^6^ PFU were spotted on O-antigen target strains as indicated (Figure 3). All phage cleared spots on their respective TSPs’ target strains except *E. coli* O26 and *E. coli* O103, consistent with previously observed lack of plaque formation. Since the reporter luciferase activity was detected in these strains, it suggests that the phage is able to adsorb and deliver the phage genome into the host cells, but unable to complete replication cycles efficiently.

Parental CBA120.NL cleared spots on all expected target strains: *Salmonella enterica* Minnesota, and *E. coli* strains O157, O77, and O78. RBP-CBA120-3 swapped the native TSP1 tropism of *Salmonella enterica* for *E. coli* O45. RBP-CBA120-5 replaced *E. coli* O78 tropism for O111 based on TSP4, while retaining O45 tropism. RBP-CBA120-6 and RBP-CBA120-9 were designed to switch *E. coli* O77 tropism for O103 and O26, respectively. However, only the expected loss of O77 was evident, and no evidence of gain of tropism for the new targets was observed. Both retained O45, O157, and O111 activity, as expected.

### 3.5. Altering Host Range Detection with Chimeric Tailspike Proteins

Although RBP-CBA120-6 and RBP-CBA120-9 were unable to generate spots or plaques on their new target strains, the expected chimeric TSP was confirmed by PCRIU_50_ in each case and increasing target-specific luciferase signal was detected during the isolation process. To check if these purified chimeric phages could be used to detect their respective target strains (Table S1), without the ability to necessarily complete phage replication, the purified stocks were subjected to luciferase-based bacterial detection assays. All target strains used in the spot assays were infected with 1.2 × 10^5^ PFU of each phage. Luciferase assays performed after a two-hour infection yielded signal on each expected bacterial strain by each phage (Figure 2). This included RBP-CBA120-6 and RBP-CBA120-9 on *E. coli* strains O103 and O26, respectively. This stands in stark contrast to the spot assay data, where those strains showed no spot clearance.

Recombinant phages RBP-CBA120-6 and RBP-CBA120-9 each contain three chimeric tailspike proteins, resulting in a modification of their bacterial strain infection and detection profile compared to the parental CBA120.NL. RBP-CBA120-6 and RBP-CBA120-9 are each able to detect four STECs: *E. coli* O45, *E. coli* O157, *E. coli* O103, and *E. coli* O111 and *E. coli* O45, *E. coli* O157, *E. coli* O26, and *E. coli* O111, respectively.

## 4. Discussion

This study demonstrates the successful engineering of chimeric tailspike proteins (TSPs) in an *Ackermannviridae* phage to modify its host range for detecting multiple STEC strains within the “Big Six” STECs [1] along with native tropism to *E. coli* O157 [23]. By replacing native TSPs of the CBA120 phage with chimeric versions incorporating receptor-binding domains from related and unrelated phages, we were able to generate recombinant phages capable of detecting four STEC serogroups each. Additionally, these changes simultaneously eliminated detection of non-STEC organisms, including *Salmonella*, *E. coli* O77, and *E. coli* O78. Based on the very high specificity of TSPs to their respective O-antigen targets and exclusivity experiments performed with *Kutterviruses* [14,22,27], off-target effects seem very unlikely. Whole genome sequencing validated the method of generating chimeric TSPs, and functional and PCR-based methods detecting the correct-sized insert and location demonstrate successful genetic engineering.

The structural conservation of TSPs, even across unrelated phage families, enabled us to design functional chimeras despite low sequence similarity. This structural approach to TSP engineering expands the toolbox for modifying phage host ranges beyond relying solely on sequence similarity. The successful generation of chimeras between *Ackermannviridae* and podoviral TSPs highlights the modularity of these proteins and the potential for mixing domains across diverse phage types. This may be the first artificial replication of the modularity observed in nature [47].

An interesting finding was the differential ability of some recombinant phages to detect versus productively infect certain STEC strains. While RBP-CBA120-6 and RBP-CBA120-9 could not form plaques or clear spots on *E. coli* O103 and O26, respectively, they were still able to likely bind to and inject their genomes into these hosts, as evidenced by the detection of luciferase signal. This suggests that these phages can absorb and initiate infection but are blocked at a later stage, possibly due to host defense mechanisms. It also demonstrates that productive infection is not necessary for phage-based detection systems, agreeing with our previous work showing bacterial detection with a replication-deficient phage [48].

The modular nature of the TSP engineering approach allowed us to iteratively expand the host range with each round of recombination. Starting from CBA120.NL with the native host range detecting only O157, we were ultimately able to expand coverage to an additional four STEC serogroups (O26, O45, O103, and O111). Critically, this approach also eliminated non-STEC cross reactivity. This showcases the potential for rationally designing “customized” phages with tailored host ranges for specific detection needs.

However, some limitations were encountered. Not all chimeric TSPs conferred the ability to productively infect their intended targets, as seen with O103 and O26. This highlights the complexity of phage–host interactions beyond just receptor binding. Factors like DNA injection, replication compatibility, and host defense mechanisms all play a role in determining productive infection. Although this issue did not prevent effective utilization of these recombinants for detection purposes, a lack of productive infection may be detrimental for other phage-based technologies, such as therapeutics. Future work could focus on understanding and overcoming these barriers to expand the utility of engineered phages. Literature searches found no satisfactory TSP candidates yet to target the remaining Big Six STEC O-antigens, O121 and O145. Future studies may incorporate O121 and O145 as new TSPs are regularly described in the literature.

The use of a luciferase reporter system proved invaluable for detecting successful infection events, even in cases where productive replication did not occur. This sensitive detection method allowed us to verify the expanded host ranges of our recombinant phages. The ability to rapidly screen for positive infections via luciferase assay also greatly facilitated the recombinant isolation process. The multiple TSPs of *Ackermannviridae* phage proved useful as each TSP could be manipulated without compromising viability of the phage on alternate host strains. This allowed chimeric production to be tracked through both loss of native TSP activity and through PCR methods without relying on phage viability on new target hosts.

In conclusion, this study demonstrates the feasibility of rationally engineering *Ackermannviridae* phages with customized host ranges for detecting multiple STEC strains. The modular nature of TSPs, combined with structure-guided design, allows for mixing and matching receptor-binding domains from diverse phage types. This approach has potential applications in developing improved phage-based diagnostics and biocontrol agents targeting foodborne pathogens. Future work could focus on further expanding the detectable range to cover the remaining “Big Six” STEC serogroups and optimizing the system for practical applications in food safety.

## Figures and Tables

**Table 1:** Source of chimeric TSPs and serogroup targets.

	Origin of Tailspike C-Terminus				Bacterial Species and Serogroups Targeted by TSP			
Phage	TSP1	TSP2	TSP3	TSP4	TSP1	TSP2	TSP3	TSP4
CBA120.NL	CBA120	CBA120	CBA120	CBA120	*S. enterica* O:21	*E. coli* O157	*E. coli* O77	*E. coli* O78
RBP-CBA120-3	Ro45lw	CBA120	CBA120	CBA120	*E. coli* O45	*E. coli* O157	*E. coli* O77	*E. coli* O78
RBP-CBA120-5	Ro45lw	CBA120	CBA120	STP55	*E. coli* O45	*E. coli* O157	*E. coli* O77	*E. coli* O111
RBP-CBA120-6	Ro45lw	CBA120	Ro103C3Iw	STP55	*E. coli* O45	*E. coli* O157	*E. coli* O103	*E. coli* O111
RBP-CBA120-9	Ro45lw	CBA120	RM10386	STP55	*E. coli* O45	*E. coli* O157	*E. coli* O26	*E. coli* O111

**Table 2:** Percent identity between tailspike proteins (TSPs) based on Needle Pairwise Sequence Alignment [32,33]. Little similarity is found with unrelated podovirus TSPs.

TSP/Accession	CBA120.TSP1	CBA120.TSP2	CBA120.TSP3	CBA120.TSP4
STP55.TSP4 *UPU15645.1*	18.0%	15.8%	18.8%	50.8%
Ro45lw.TSP *YP_009818296.1*	15.5%	15.2%	17.5%	11.6%
Ro103C3Iw.TSP *QDH94159.1*	18.6%	14.6%	20.9%	14.7%
RM10386.TSP *WP_038987731.1*	16.7%	13.6%	13.7%	13.1%

## Data Availability

Data are contained within the article or Supplementary Material. All other sequences are publicly available. Availability of the engineered bacteriophages described in this study and those generated previously may require a material transfer agreement covering potential commercial applications.

## Supplementary Materials

The following supporting information can be downloaded at https://www.mdpi.com/article/10.3390/v17121614/s1: Figure S1: Alignments of TSP operons from *Kutterviruses* CBA120 and STP55 [14,32,33], Table S1: List of bacterial strains used in this study, Table S2: Chimeric TSP components, Table S3: List of plasmids used in this study, Figure S2: O45-specific TSP cloning and alignments [37,39,40], Figure S3: O111-specific chimera cloning and alignments, Figure S4: RBP-CBA120-6 O103-specific chimera cloning, alignments and PCR [39], Table S4: Primers used to detect recombinants via PCR, Figure S5: RBP-CBA120-6 plaque screen for loss of O77 tropism, Figure S6: Luciferase-based infection assay to detect O103 activity, Figure S7: RBP-CBA120-9 O26-specific chimera cloning, alignments and PCR, Figure S8: Luciferase-based infection assay to detect O26 activity, Table S5: Luciferase-based tropism tests raw data, and Table S6: AlphaFold2 prediction confidence values.
