# Supplementary material for: Generating STEC-Specific Ackermannviridae Bacteriophages Through Tailspike Protein Chimerization
Source: Viruses. 2025 Dec 14;17(12):1614. doi: 10.3390/v17121614 (PMC12737597; doi:10.3390/v17121614)
Supplement: Supplementary file 1 [file viruses-17-01614-s001.zip › viruses-3991282-supplementary.pdf]

Supplementary Materials

Generating STEC-specific *Ackermannviridae* Bacteriophages through Tailspike Protein Chimerization

Jose Gil [1], John Paulson [2], Henriett Zahn [2], Matthew Brown [3], Minh M. Nguyen [2] and Stephen Erickson [2,*]

[1] Labcorp, Calabasas, CA 91301, USA; gilj@labcorp.com
[2] Labcorp, New Brighton, MN 55112, USA; paulsoj@labcorp.com (J.P.); zahnh@labcorp.com (H.Z.); nguyem5@labcorp.com (M.M.N.)
[3] Labcorp, Burlington, NC 27215, USA; matthewjudebrown@gmail.com
* Correspondence: erickss@labcorp.com

Table S1. List of Bacterial Strains Used in This Study. Strains were obtained from either the American Type Culture Collection (ATCC), Michigan State University STEC Center (MSU) and the United States Department of Agriculture (USDA)

**Bacteria**	**Serogroup**	**Stx1/Stx2/eae**	**Strain ID**	**Source**
Escherichia coli	O26:H11	Stx1+/stx2+/eae+	BAA-2205	ATCC
Escherichia coli	O26:H11	Stx1+/stx2+/eae+	BAA-2196	ATCC
Escherichia coli	O26:H4	Neg	BAA-2212	ATCC
Escherichia coli	O26:H11	Stx1+/stx2+/eae+	May063	USDA
Escherichia coli	O45:H2	Stx1+/stx2+/eae+	BAA-2193	ATCC
Escherichia coli	O77	NA	23537	ATCC
Escherichia coli	O78	NA	ECOR70	MSU
Escherichia coli	O103:H2	Stx1+/stx2+/eae+	BAA-2210	ATCC
Escherichia coli	O103	Neg	BAA-2214	ATCC
Escherichia coli	O111:H8	Stx1+/eae+	BAA-2201	ATCC
Escherichia coli	O157:H7	Stx1-/stx2-/eae+	43888	ATCC
Salmonella enterica	O:21		52329.1	USDA

Table S2. Chimeric TSP Components. Break down of the chimeric TSP components by amino acid

	**N-term Region**		**C-term Region**	
**Chimeric Phage**	**CBA120 TSP**	**Amino Acids**	**Donor Phage**	**Amino Acids**
RBP-CBA120-3	TSP1	1-163	Ro45lw	156-669
RBP-CBA120-5	TSP4	1-490	STP55	491-1011
RBP-CBA120-6	TSP3	1-197	Ro103C3lw	237-696
RBP-CBA120-9	TSP3	1-168	RM10386	120-707

Supplementary Materials

Table S3. List of Plasmids Used in This Study. All homologous recombination (HR) plasmids used in this study to generate chimeric TSPs were synthesized by GeneWiz (GeneWiz, South Plainfield, NJ, USA) and verified by Sanger sequencing by the manufacturer.

**Plasmid**	**Source TSP**	**Target TSP**	**O-Antigen**	**Resulting Phage**
pUCGW.HR.CBA120.TSP1-Ro45.TSP	Ro45lw	TSP1	O45	RBP-CBA120-3
pUCGW.HR.CBA120-STP55.TSP4	STP55	TSP4	O111	RBP-CBA120-5
pUCGW.HR.CBA120.TSP3-Ro103.TSP	Ro103C3lw	TSP3	O103	RBP-CBA120-6
pUCGW.HR.CBA120.TSP3-RM10386.TSP	RM10386	TSP3	O26	RBP-CBA120-9

Table S4. Primers Used to Detect Recombinants via PCR. Forward and reverse primers to detect recombinant phages RBP-CBA120-6 and RBP-CBA120-9. One primer falls within the donor TSP sequence, while the other primer falls in the wild-type sequence past the flanking sequence identity arms for homologous recombination present in the plasmid.

	Forward Recombinant	Reverse Recombinant	Reverse Wild-Type
RBP-CBA120-6	CGACCCGTTCTCTATTCATCTC	TTGTGTGGCCTTACCACTATC	TACGCCAGTGACAGGATAGA
RBP-CBA120-9	CACAGTGAACTCCATCGGATAC	TCCCAGCAGATACAGAAGAAATG	none

Table S5. Luciferase Based Tropism Tests Raw Data. Raw data in RLU/s from GloMax Navigator luminometer reads of recombinant phage tropism tests (Figure 3).

Strain	CBA120.NL	RBP-CBA120-5	RBP-CBA120-6	RBP-CBA120-9
TSB control	73	104	80	98
S. enterica 52329.1	1,433,667,840	83	99	110
O157 43888	491,886,976	519,890,016	248,324,272	382,513,792
O77 23537	572,767,424	863,817,408	67	85
O78 ECOR70	684,150,784	83	68	102
O45 BAA-2193	80	1,799,767,808	1,671,713,152	2,681,891
O111 BAA-2201	60	66,730,820	57,895,416	36,233,972
O103 BAA-2210	66	88	957,115	82
O26 BAA-2196	58	101	91	3,978,650

Supplementary Materials

Table S6. AlphaFold2 Prediction Confidence Values. Average pLDDT confidence values of AlphaFold2 structural predictions.

Predicted Structure	Average pLDDT
CBA120.TSP2	77.9%
CBA120.TSP3	89.0%
CBA120.TSP4	74.2%
Ro45lw.TSP	93.2%
STP55.TSP4	75.7%
Ro103C3lw.TSP	87.3%
RM10386.TSP	94.9%
CBA120.TSP1-Ro45lw.TSP	95.0%
CBA120.TSP3-Ro103C3lw.TSP	90.2%
CBA120.TSP3-RM10386.TSP	95.4%
CBA120-STP55.TSP4	78.8%

Figure S1. Alignments of TSP operons from *Kutterviruses* CBA120 and STP55

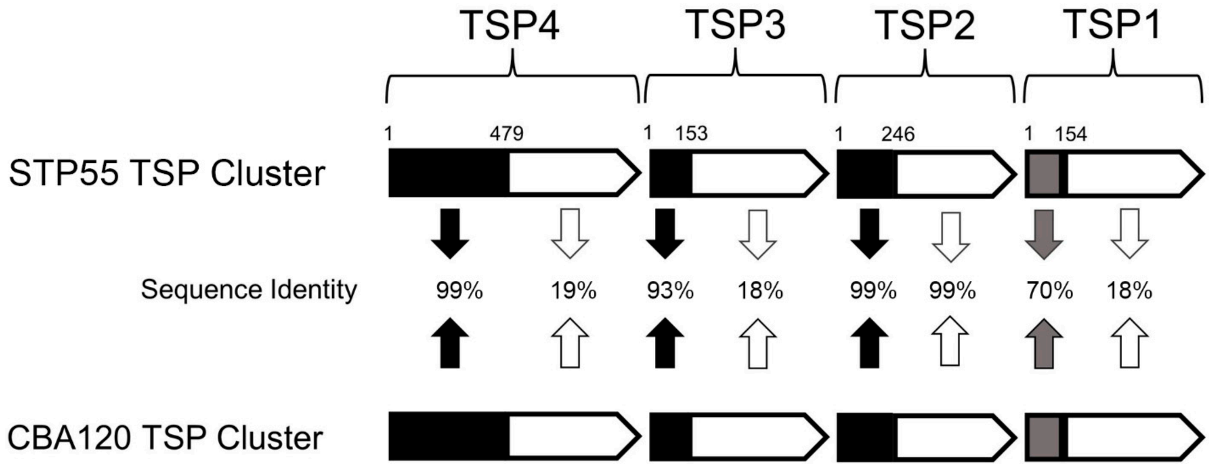

Figure S1. The genomes of the *Kuttervirus* TSP operon share high sequence identity in the N-terminal domains. Delineation between the N-terminal head and C-terminal body previously determined by sequence alignment [1]. Comparison of N- and C-terminal regions for each TSP were performed using EMBOSS Needle Pairwise Alignment [2,3]. Amino acid sequence percent identity between each TSP's N or C terminal domain are compared between CBA120 and STP55.

Supplementary Materials

Figure S2. O45 Specific TSP Cloning and Alignments

A

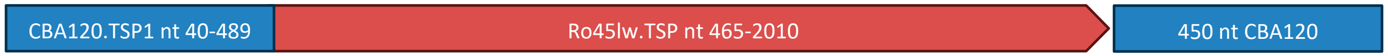

B

```
CBA120.TSP1              MNEMFSQGGKGSTGILTNKQAVARHFGVKQSEVVYFSVGVDLGGYKVIYDKETQRAYSLPVGIASGTTAVSLSTAAVLVHSAGSVDLGSLAVSREEYVTL
                         ||||||||||||||||||||||||||||||||||||||||||||||||||||||||||||||||||||||||||||||||||||||||||||||||||||
CBA120.TSP1-Ro45lw.TSP_  MNEMFSQGGKGSTGILTNKQAVARHFGVKQSEVVYFSVGVDLGGYKVIYDKETQRAYSLPVGIASGTTAVSLSTAAVLVHSAGSVDLGSLAVSREEYVTL
                                    ||        |               |                                                            |
Ro45lw.TSP                       MANSTLTKFPSGRVQYKINFPYLARAFVVVTLVVSTDPTKDKVLVVGNDYRFLNETTLEILADQTGFDIIQIHRFTSTELVVDFRDGSVLTA

CBA120.TSP1              PGSFDSGSTLNVKNELLTYTDGKYRWDGILPKTVAPGSTPASTGGVGLGAWISVGDASLRTQLANGDGSLIGIHPQGTLNNVLTVRTPEQYNAVGDGIAD
                         |||||||||||||||||||||||||||||||||||||||||||||||||||||||||||||||    |             |         |
CBA120.TSP1-Ro45lw.TSP_  PGSFDSGSTLNVKNELLTYTDGKYRWDGILPKTVAPGSTPASTGGVGLGAWISVGDASLRTQLRSDTGLSMIHRKLPDIGAVNVGMDALFGNMLNPVDFG
                                       |                             |          |  ||  ||||||||||||||||||||||||||||||||||||||
Ro45lw.TSP               SDLSTAEIQAIHIAEEGRDQSINLASGYTEEAKKYADEASDALEGIKDIVQDGNFDTTLRQDLRSDTGLSMIHRKLPDIGAVNVGMDALFGNMLNPVDFG

CBA120.TSP1              DTSKLKEMLSDINNVPETLPDAAAVNSYMEQVAVKIDLTKLYRFTETLYIPPGVSIEIPTSNFFTRECKQGLFYDPVDKNTAAISLMVYRKQPDGSYKLN
                                                           |       |      |     |
CBA120.TSP1-Ro45lw.TSP_  ADPTGSVDSYAALQKWADACTSRPWAYAFISGIFKVSQPVTFRNIRGMTIYGHCYIYPTFDQGDYVVGFCNGAGLRIHGRFEVSGQNKVSIKTGVKIWSD
                         ||||||||||||||||||||||||||||||||||||||||||||||||||||||||||||||||||||||||||||||||||||||||||||||||||||
Ro45lw.TSP               ADPTGSVDSYAALQKWADACTSRPWAYAFISGIFKVSQPVTFRNIRGMTIYGHCYIYPTFDQGDYVVGFCNGAGLRIHGRFEVSGQNKVSIKTGVKIWSD

CBA120.TSP1              KDVDYYPTGLDIDNGDAITCARKIDINNLNLITAPGVKVGVKWIGGAGCTTKGLSIGENTGSDITTARLPRVGLLQSASWGSIHENLRILYKTQGAVFID
                                                                                |                           |  |
CBA120.TSP1-Ro45lw.TSP_  MNPNGFSFSYFYGMCVSDANPGITLGDTRYANALLSELSFIGGYTVGTPCSIKAIGTQCYFNVIGFDAVTGGAGDLAQVTPYTYHLKGAQMKVIGGEIQH
                         ||||||||||||||||||||||||||||||||||||||||||||||||||||||||||||||||||||||||||||||||||||||||||||||||||||
Ro45lw.TSP               MNPNGFSFSYFYGMCVSDANPGITLGDTRYANALLSELSFIGGYTVGTPCSIKAIGTQCYFNVIGFDAVTGGAGDLAQVTPYTYHLKGAQMKVIGGEIQH

CBA120.TSP1              SNGGAAVNNAYISRLGNTNGELEQAVYKPAGFTEVGDVAVTQFAGSEVKFNSPIIEQASFDFVHAGRDTDSYGLFMVDKPHIESSGGKKKHSFYLINTSS
                                            |     |       ||    |       |               |     |      |        |
CBA120.TSP1-Ro45lw.TSP_  NDSIVGAAILLEPIVDPVHGNTYGNVTVDQSHFEVASTWVMVANLDGVPNPISHRSSVTLIGLHGYHNQDNGPVVLVHESADDYRGTIVTRDISMYRGDD
                         ||||||||||||||||||||||||||||||||||||||||||||||||||||||||||||||||||||||||||||||||||||||||||||||||||||
Ro45lw.TSP               NDSIVGAAILLEPIVDPVHGNTYGNVTVDQSHFEVASTWVMVANLDGVPNPISHRSSVTLIGLHGYHNQDNGPVVLVHESADDYRGTIVTRDISMYRGDD

CBA120.TSP1              NVTLSGVGLSGQDPDLDSMYFLKNCPETARNVVRGQMPISGVKLVRGTGNYPTLVLDCTNMGSQFQFGEVGDIFYIKDVVGVKADTLYIDPVNGNNYNWG
                                         |                         |               |                          |       |
CBA120.TSP1-Ro45lw.TSP_  SPDRVNMNIQARNAYVDYDRKGFGKGFVRGLQAVQGGVLKFEKSTICIAQNSVGQTHTTAPNNVLYTQFIANGDTDRWSSNYNPATGEFTVPAGGLKDVH
                         ||||||||||||||||||||||||||||||||||||||||||||||||||||||||||||||||||||||||||||||||||||||||||||||||||||
Ro45lw.TSP               SPDRVNMNIQARNAYVDYDRKGFGKGFVRGLQAVQGGVLKFEKSTICIAQNSVGQTHTTAPNNVLYTQFIANGDTDRWSSNYNPATGEFTVPAGGLKDVH
```

Supplementary Materials

```
CBA120.TSP1                 TNGTKPIRELTNIAKICQLFRCKSVYLNAGESVITSNTELPMVVFEGPGSLKANSGSSFLIKAGGTLSLIGLSGISTDGGHMFRVSTVEKVNIHTNCSVN
                               |                                   |         | |   |       |  |
CBA120.TSP1-Ro45lw.TSP_     VEATVEVASSSQVTMAVYVDNDRKNLVQTLPVAGNTSAYLGHLPAGAKISVKCQLGTGSGASTGAGLDRMVITASNY
                            |||||||||||||||||||||||||||||||||||||||||||||||||||||||||||||||||||||||||||||
Ro45lw.TSP                  VEATVEVASSSQVTMAVYVDNDRKNLVQTLPVAGNTSAYLGHLPAGAKISVKCQLGTGSGASTGAGLDRMVITASNY

CBA120.TSP1                 AGAAYVVLSEVQGNIEYRQLFYSVNCSKYIGATAGQTIAGIMVKTATRPTGIDAAPVDGNVSLTYKIIES

CBA120.TSP1-Ro45lw.TSP_

Ro45lw.TSP
```

C

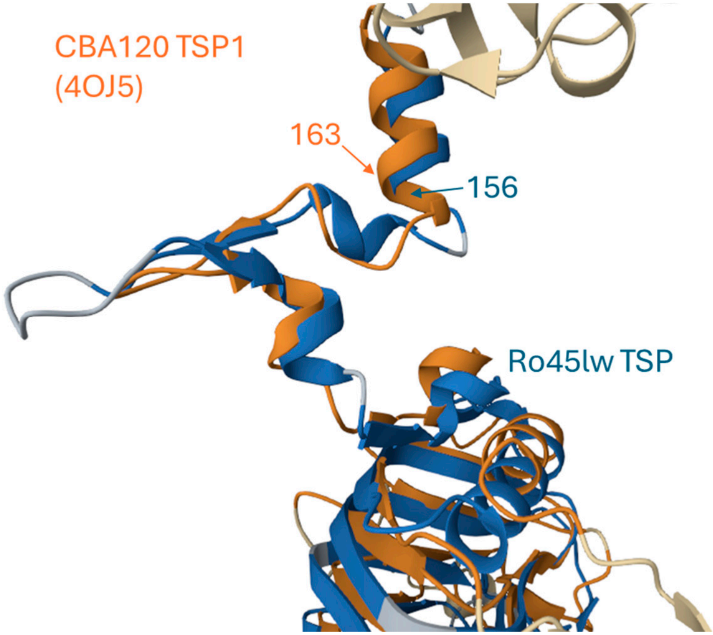

Figure S2: A) Homologous recombination (HR) construct from pUCGW.HR.CBA120.TSP1-Ro45TSP to insert nucleotides 465 – 2010 of Ro45lw TSP into the CBA120 genome, replacing the C-terminal domains of TSP1 (ORF210), with 450 nucleotides (nt) of wild-type CBA120 sequence flanking the insert. Construct created in A Plasmid Editor [4]. B) The amino acid sequences of the recipient, donor and chimeric proteins were aligned based on splice site used, with native CBA120 TSP1 shown in green and Ro45lw TSP in red. C) RCSB Pairwise Structure Alignment of CBA120 TSP1 (brown) crystal structure (4OJ5) [5] and AlphaFold2 [6] prediction of Ro45lw TSP (blue), with amino acids flanking splice indicated.

Supplementary Materials

Figure S3. O111 Specific Chimera Cloning and Alignments

A

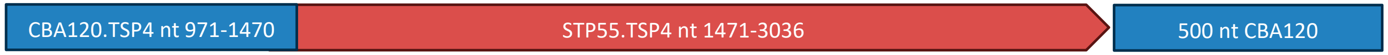

B

```
# Identity:     601/1184 (50.8%)
# Similarity:   667/1184 (56.3%)
# Gaps:         319/1184 (26.9%)
# Score: 2555.5
CBA120_TSP4         1 MANKPTQPLFPLGLETSESSNIKGFNNSGTIEHSPGAVMTFPEDTEVTGL     50
                      ||||||||||||||||||||||||||||||||||||||||||||||||||
STP55.TSP4          1 MANKPTQPLFPLGLETSESSNIKGFNNSGTIEHSPGAVMTFPEDTEVTGL     50
CBA120_TSP4        51 PSSVRYNPDSDEFEGYYENGGWLSLGGGGIRWETLPHAPSSNLLEGRGYL    100
                      ||||||||||||||||||||||||||||||||||||||||||||||||||
STP55.TSP4         51 PSSVRYNPDSDEFEGYYENGGWLSLGGGGIRWETLPHAPSSNLLEGRGYL    100
CBA120_TSP4       101 INNTTGTSTVVLPSPTRIGDSVTICDAYGKFATYPLTVSPSGNNLYGSTE    150
                      ||||||||||||||||||||||||||||||||||||||||||||||||||
STP55.TSP4        101 INNTTGTSTVVLPSPTRIGDSVTICDAYGKFATYPLTVSPSGNNLYGSTE    150
CBA120_TSP4       151 DMAITTDNVSATFTWSGPEQGWVITSGVGLGQGRVYSREIFTQILASETS    200
                      ||||||||||||||||||||||||||||||||||||||||||||||||||
STP55.TSP4        151 DMAITTDNVSATFTWSGPEQGWVITSGVGLGQGRVYSREIFTQILASETS    200
CBA120_TSP4       201 AVTLNTPPTIVDVYADGKRLAESKYSLDGNVITFSPSLPASTELQVIEYT    250
                      ||||||||||||||||||||||||||||||||||||||||||||||||||
STP55.TSP4        201 AVTLNTPPTIVDVYADGKRLAESKYSLDGNVITFSPSLPASTELQVIEYT    250
CBA120_TSP4       251 PIQLGNGGGSGSSTITWVYNGGSAIGGETEITLDIVVDDVPAIDINGSRQ    300
                      ||||||||.|.|||||||||||||||||||||||:|||||||||||||||
STP55.TSP4        251 PIQLGNGGDSSSSTITWVYNGGSAIGGETEITLDVVVDDVPAIDINGSRQ    300
CBA120_TSP4       301 YKNLGFTFDPLTSKITLAQELDAEDEVVVIINGTPNIYNQIDYTLREVAR    350
                      ||||||||||||||||||||||||||||||||||||||||||||||||||
STP55.TSP4        301 YKNLGFTFDPLTSKITLAQELDAEDEVVVIINGTPNIYNQIDYTLREVAR    350
CBA120_TSP4       351 VTNVKDTEVIYFSVGAVLSGYKVIYDKVTQRSYFIPELPTGTTAVSLSSS    400
                      ||||||||||||||||||||||||||||||||||||||||||||||||||
STP55.TSP4        351 VTNVKDTEVIYFSVGAVLSGYKVIYDKVTQRSYFIPELPTGTTAVSLSSS    400
CBA120_TSP4       401 AILVHSAGSVDLGALAVSREEYVTLSGTFDSGAVINTKNELLTHTDGKYR    450
                      |:||||||||||||||||||||||||||||||||||||||||||||||||
STP55.TSP4        401 AVLVHSAGSVDLGALAVSREEYVTLSGTFDSGAVINTKNELLTHTDGKYR    450
CBA120_TSP4       451 WDGTLPKTVAAGSTPATTGGVGSGAWLSVGDASLKSNLNKPNGLSYIGTV    500
                      ||||||||||.||||.||||||.|||:|||||||:|:|.|.:|.:.|.:|
STP55.TSP4        451 WDGTLPKTVAPGSTPITTGGVGVGAWISVGDASLRSDLLKDDGYTLIPSV    500
CBA120_TSP4       501 --------SSVSELSSIAGLIGDSII---LDSYVDGFNLGGGVMVAVNSD    539
                              ..:....::.|...||.|   |:|......|||...|:..||
STP55.TSP4        501 QIQQWRAEGDIRGWGAVDGGFNDSAILAALNSDSPSIKLGGRGFVSKVSD    550
```

Supplementary Materials

```
CBA120_TSP4       540 T--------VVDNIVTFQGNGVVWKRKLFNGV-------ADVYEAGYTGT    574
                      |        :....:.||..|...:.|  :|:       |.|.:...|||
STP55.TSP4        551 TINHKSNKVIHSGSLNFQFVGGTQQEK--SGILMANIANAKVIDVDITGT    598
CBA120_TSP4       575 GDLAIFINKINAVGFDCIVPVSGEITTPIIFDIAKGALIGKNKCTLIESA    624
                      .|     ..|...|...||      |....:.||...||.|  :|.:   .
STP55.TSP4        599 LD-----GGIRGYGGSNIV-----IDGVSVHDIGVSALTG--ECGM---G    633
CBA120_TSP4       625 SATGDYYLTIVNTDTDYTNRDVINATALMTGVSFVGKGTRKLAIGGSTSG    674
                      ...|||        ..|.|:                           |.|
STP55.TSP4        634 IWFGDY--------AHYENQ---------------------------TDG    648
```

C

```
CBA120.TSP4         MANKPTQPLFPLGLETSESSNIKGFNNSGTIEHSPGAVMTFPEDTEVTGLPSSVRYNPDSDEFEGYYENGGWLSLGGGGIRWETLPHAPSSNLLEGRGYL
                    ||||||||||||||||||||||||||||||||||||||||||||||||||||||||||||||||||||||||||||||||||||||||||||||||||||
CBA120-STP55.TSP4   MANKPTQPLFPLGLETSESSNIKGFNNSGTIEHSPGAVMTFPEDTEVTGLPSSVRYNPDSDEFEGYYENGGWLSLGGGGIRWETLPHAPSSNLLEGRGYL
                    ||||||||||||||||||||||||||||||||||||||||||||||||||||||||||||||||||||||||||||||||||||||||||||||||||||
STP55.TSP4          MANKPTQPLFPLGLETSESSNIKGFNNSGTIEHSPGAVMTFPEDTEVTGLPSSVRYNPDSDEFEGYYENGGWLSLGGGGIRWETLPHAPSSNLLEGRGYL

CBA120.TSP4         INNTTGTSTVVLPSPTRIGDSVTICDAYGKFATYPLTVSPSGNNLYGSTEDMAITTDNVSATFTWSGPEQGWVITSGVGLGQGRVYSREIFTQILASETS
                    ||||||||||||||||||||||||||||||||||||||||||||||||||||||||||||||||||||||||||||||||||||||||||||||||||||
CBA120-STP55.TSP4   INNTTGTSTVVLPSPTRIGDSVTICDAYGKFATYPLTVSPSGNNLYGSTEDMAITTDNVSATFTWSGPEQGWVITSGVGLGQGRVYSREIFTQILASETS
                    ||||||||||||||||||||||||||||||||||||||||||||||||||||||||||||||||||||||||||||||||||||||||||||||||||||
STP55.TSP4          INNTTGTSTVVLPSPTRIGDSVTICDAYGKFATYPLTVSPSGNNLYGSTEDMAITTDNVSATFTWSGPEQGWVITSGVGLGQGRVYSREIFTQILASETS

CBA120.TSP4         AVTLNTPPTIVDVYADGKRLAESKYSLDGNVITFSPSLPASTELQVIEYTPIQLGNGGGSGSSTITWVYNGGSAIGGETEITLDIVVDDVPAIDINGSRQ
                    ||||||||||||||||||||||||||||||||||||||||||||||||||||||||||||||||||||||||||||||||||||||||||||||||||||
CBA120-STP55.TSP4   AVTLNTPPTIVDVYADGKRLAESKYSLDGNVITFSPSLPASTELQVIEYTPIQLGNGGGSGSSTITWVYNGGSAIGGETEITLDIVVDDVPAIDINGSRQ
                    |||||||||||||||||||||||||||||||||||||||||||||||||||||||||| | ||||||||||||||||||||||| |||||||||||||||
STP55.TSP4          AVTLNTPPTIVDVYADGKRLAESKYSLDGNVITFSPSLPASTELQVIEYTPIQLGNGGDSSSSTITWVYNGGSAIGGETEITLDVVVDDVPAIDINGSRQ

CBA120.TSP4         YKNLGFTFDPLTSKITLAQELDAEDEVVVIINGTPNIYNQIDYTLREVARVTNVKDTEVIYFSVGAVLSGYKVIYDKVTQRSYFIPELPTGTTAVSLSSS
                    ||||||||||||||||||||||||||||||||||||||||||||||||||||||||||||||||||||||||||||||||||||||||||||||||||||
CBA120-STP55.TSP4   YKNLGFTFDPLTSKITLAQELDAEDEVVVIINGTPNIYNQIDYTLREVARVTNVKDTEVIYFSVGAVLSGYKVIYDKVTQRSYFIPELPTGTTAVSLSSS
                    ||||||||||||||||||||||||||||||||||||||||||||||||||||||||||||||||||||||||||||||||||||||||||||||||||||
STP55.TSP4          YKNLGFTFDPLTSKITLAQELDAEDEVVVIINGTPNIYNQIDYTLREVARVTNVKDTEVIYFSVGAVLSGYKVIYDKVTQRSYFIPELPTGTTAVSLSSS

CBA120.TSP4         AILVHSAGSVDLGALAVSREEYVTLSGTFDSGAVINTKNELLTHTDGKYRWDGTLPKTVAAGSTPATTGGVGSGAWLSVGDASLKSNLNKPNGLSYIGTV
                    ||||||||||||||||||||||||||||||||||||||||||||||||||||||||||||||||||||||||||||||||||||||||||  |   |  |
CBA120-STP55.TSP4   AILVHSAGSVDLGALAVSREEYVTLSGTFDSGAVINTKNELLTHTDGKYRWDGTLPKTVAAGSTPATTGGVGSGAWLSVGDASLKSNLNKDDGYTLIPSV
                    | |||||||||||||||||||||||||||||||||||||||||||||||||||||||||| |||| |||||| ||| ||||||| | | |||||||||||
STP55.TSP4          AVLVHSAGSVDLGALAVSREEYVTLSGTFDSGAVINTKNELLTHTDGKYRWDGTLPKTVAPGSTPITTGGVGVGAWISVGDASLRSDLLKDDGYTLIPSV
```

```
CBA120.TSP4             SSVSELSSIAGLIGDSIILDSYVDGFNLGGGVMVAVNSDTVVDNIVTFQGNGVVWKRKLFNGVADVYEAGYTGTGDLAIFINKINAVGFDCIVPVSGEIT
                                                                                 |                           |      |   |
CBA120-STP55.TSP4       QIQQWRAEGDIRGWGAVDGGFNDSAILAALNSDSPSIKLGGRGFVSKVSDTINHKSNKVIHSGSLNFQFVGGTQQEKSGILMANIANAKVIDVDITGTLD
                        ||||||||||||||||||||||||||||||||||||||||||||||||||||||||||||||||||||||||||||||||||||||||||||||||||||
STP55.TSP4              QIQQWRAEGDIRGWGAVDGGFNDSAILAALNSDSPSIKLGGRGFVSKVSDTINHKSNKVIHSGSLNFQFVGGTQQEKSGILMANIANAKVIDVDITGTLD

CBA120.TSP4             TPIIFDIAKGALIGKNKCTLIESASATGDYYLTIVNTDTDYTNRDVINATALMTGVSFVGKGTRKLAIGGSTSGEVSELRISNCGFISTAGIEFLDNAYR
                          |         |       |     ||     |   |                     |        |             |
CBA120-STP55.TSP4       GGIRGYGGSNIVIDGVSVHDIGVSALTGECGMGIWFGDYAHYENQTDGLLIQNCRINNIGGVGIMRGDGIGVYNAKNFTIRHNNTVTVNRMGISTGSDCT
                        ||||||||||||||||||||||||||||||||||||||||||||||||||||||||||||||||||||||||||||||||||||||||||||||||||||
STP55.TSP4              GGIRGYGGSNIVIDGVSVHDIGVSALTGECGMGIWFGDYAHYENQTDGLLIQNCRINNIGGVGIMRGDGIGVYNAKNFTIRHNNTVTVNRMGISTGSDCT

CBA120.TSP4             ILFDKCALSRSFTNSVIFNSPANSGEVIKFNHCWMVDNGGPFTFKNGQFIFDSCSLPAGKKSGYFDPVVALSDNATTVFTNGNIEYQPGQSFVGFTVDGS
                                        |   |                 |                            |      |    |                 |
CBA120-STP55.TSP4       YFKVHSNYIGDTLLAAIDIEPDSVYTASNGKVYNNTIVGFASRYFAEGAGVGQTFGIDIHANTSYIKVYGNTLSAGLYGTEAFHIGNNADEIEITDNDLI
                        ||||||||||||||||||||||||||||||||||||||||||||||||||||||||||||||||||||||||||||||||||||||||||||||||||||
STP55.TSP4              YFKVHSNYIGDTLLAAIDIEPDSVYTASNGKVYNNTIVGFASRYFAEGAGVGQTFGIDIHANTSYIKVYGNTLSAGLYGTEAFHIGNNADEIEITDNDLI

CBA120.TSP4             SRLSISDSTILLPNDYSTVPIVNNGDGVVSLNNCSLPLYGSTTIATGFATRQLIGGLSKKIMSRGCYPRAGFITSNWNLGCIVSPYINSVSNGSGQFENI
                                 |          |  |     |          |                                                         |
CBA120-STP55.TSP4       GGAVVIPLFIKTYDGSGSKHIKINRNRAKSTCSSFADVAMSEDVYISENVFSGNSSNDSYFLRASTIAGLNVDYNRSSNTTNFIKSGDAGNTSNVKVTNN
                        ||||||||||||||||||||||||||||||||||||||||||||||||||||||||||||||||||||||||||||||||||||||||||||||||||||
STP55.TSP4              GGAVVIPLFIKTYDGSGSKHIKINRNRAKSTCSSFADVAMSEDVYISENVFSGNSSNDSYFLRASTIAGLNVDYNRSSNTTNFIKSGDAGNTSNVKVTNN

CBA120.TSP4             SNWTLSQTGTDVVTVTTGNDVPNDLMFSTSFVLSVPTVGAAANFTQTIIDCEPGRYFQLGFWAKNTTTTLASIRFLDQQGNAVADSIGYNIPVGNTFNFY
                           ||                                                            |                               |
CBA120-STP55.TSP4       NISTLLDGIDILTSGSLAGFIASGNTILCPASNKGISLEVYGAGSISDLRLRGNIIYNATTKIYVTPSATGWDMLTKNSRTDLTGVQNGTQLFELSRNRV
                        ||||||||||||||||||||||||||||||||||||||||||||||||||||||||||||||||||||||||||||||||||||||||||||||||||||
STP55.TSP4              NISTLLDGIDILTSGSLAGFIASGNTILCPASNKGISLEVYGAGSISDLRLRGNIIYNATTKIYVTPSATGWDMLTKNSRTDLTGVQNGTQLFELSRNRV

CBA120.TSP4             ALVDCVPPGAYKAEINFNVSSIVGGIAIHNVIYGLI

CBA120-STP55.TSP4       TQFLNGTWDDS
                        |||||||||||
STP55.TSP4              TQFLNGTWDDS
```

Figure S3: A) Homologous recombination construct to insert nucleotides 1471 – 3036 of STP55 TSP4 into the CBA120 genome, replacing the C-terminal domains of TSP4 (ORF213), construct created in A Plasmid. B) N-terminal homology demonstrated by Needle Pairwise Alignment of amino acid sequences of both TSP4s, with the neck region highlighted in blue. C) The amino acid sequences of the recipient, donor, and chimeric proteins were aligned based on splice site used, with native CBA120 TSP4 shown in green and STP55 TSP4 in red.

Supplementary Materials

Figure S4. RBP-CBA120-6 O103 Specific Chimera Cloning, Alignments and PCR

A

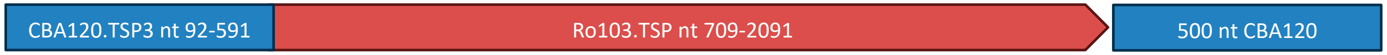

B

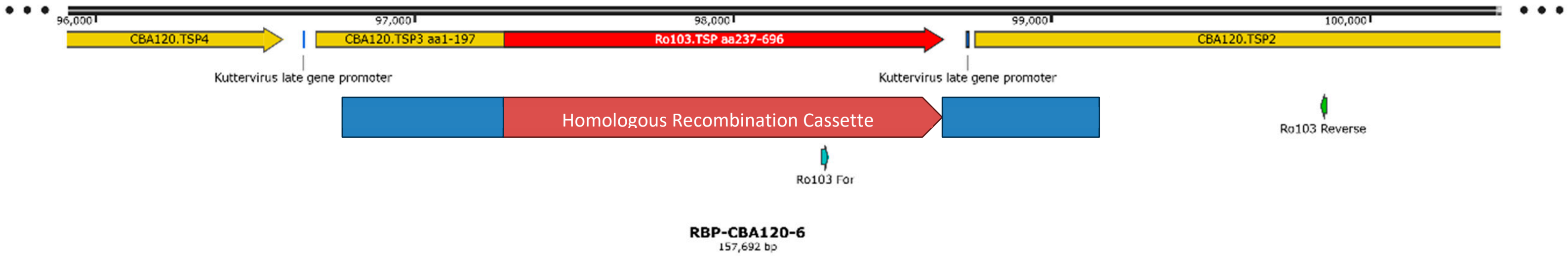

C

```
CBA120.TSP3                                                                                               MISQFNQPRGSTSIEVNKQSIARNFG
CBA120.TSP3-Ro103C3Iw.TSP                                                                                 MISQFNQPRGSTSIEVNKQSIARNFG
Ro103C3lw.TSP               MANTTITQFPTGQTQYKINFDYLARTFVVVTLINSADQADNKVLTVGNDYRFITATTIELLADQTGFDIVQIHRFTGTGLLVSFRDGSVLTANDLTTSEL

CBA120.TSP3                 VKEDEVIYFTAGIDLSGFKVIYDESTQRAYSLPFGIVSGTTAISLDERAILTHSAGSVDLGELAVSREEYVTLPGSFNFGHTINVKNELLVHDDKKYRWD
CBA120.TSP3-Ro103C3Iw.TSP   VKEDEVIYFTAGIDLSGFKVIYDESTQRAYSLPFGIVSGTTAISLDERAILTHSAGSVDLGELAVSREEYVTLPGSFNFGHTINVKNELLVHDDKKYRWD
Ro103C3lw.TSP               QAIHIAEEGRDQTSGLAKQYADQAIAAGTDAQSILDNIIKMGKNGYTPVGSFEAGATVNLQNDAVQFGTGAAITHWRWEGTLPKVVPPNSTPTSTGGIGH

CBA120.TSP3                 GSLPKVVAAGSTPDSSGGVGLGAWLSVGDAALRAELNTKVSDGTFPATIKYKYGLPSVIDGAIYRTVQDKLDDFVFLEDFGGKDDAGSTDNSIAFRKAFA
CBA120.TSP3-Ro103C3Iw.TSP   GSLPKVVAAGSTPDSSGGVGLGAWLSVGDAALRAELSGTLRVVDSVKDLESLVPQQDGEIVIARWAAYPKLVGVNCGPVGGGMFIAYNDNPAVPKDGGFF
Ro103C3lw.TSP               GKWIDVTDATLRGALAQLTGASLVMTSNGKTVEERLSGTLRVVDSVKDLESLVPQQDGEIVIARWAAYPKLVGVNCGPVGGGMFIAYNDNPAVPKDGGFF
```

Supplementary Materials

```
CBA120.TSP3                   SGARKIRLRGSGVYGMATRDIELPAKYEIIGNAKNPEIKYLGTDTSFTMFTLTGSGPASNQWKQGGMFRDLIISSDVKINWMLGRHVQNLDYDRVFFYNS
                                     |                          |                                                       |
CBA120.TSP3-Ro103C3Iw.TSP     VDSPSPTLKWRRMAENTGYYPVAWWGVIPDHYTDNADNITRAHNFAKRAWRVLQYDFGTYLTSKAVPIYSRMGIKGTGRADGTVIAKTTNDAFNLLKADG
                              ||||||||||||||||||||||||||||||||||||||||||||||||||||||||||||||||||||||||||||||||||||||||||||||||||||
Ro103C3lw.TSP                 VDSPSPTLKWRRMAENTGYYPVAWWGVIPDHYTDNADNITRAHNFAKRAWRVLQYDFGTYLTSKAVPIYSRMGIKGTGRADGTVIAKTTNDAFNLLKADG

CBA120.TSP3                   ATVLNNYHYVNFTRCERWGSAFIGRADLNTIQFISESPKFHLCFSSGSPIDVWDTADLAITKCTMFAGDYAVRTRVTQKQVTAPDLFAGYPVLITCSVFD
                                                                    |                                                    |  |
CBA120.TSP3-Ro103C3Iw.TSP     TVGEAVDALAVMVPDAYDRASPYMDSFCIHGRIEDIMFKRNGLSEANYNTMRPQYGLFMNKGGSPVLKHTNFEGAYIGIRAYVCFSSTISAVATTNWRGK
                              ||||||||||||||||||||||||||||||||||||||||||||||||||||||||||||||||||||||||||||||||||||||||||||||||||||
Ro103C3lw.TSP                 TVGEAVDALAVMVPDAYDRASPYMDSFCIHGRIEDIMFKRNGLSEANYNTMRPQYGLFMNKGGSPVLKHTNFEGAYIGIRAYVCFSSTISAVATTNWRGK

CBA120.TSP3                   AVRGHAWDLEGSVYSTITGNLVSAGRDTNSHGAYIKGGRSLSLTGNVFTYCGNYGLVLEDVQQSGFVGNVFNGNKTGGLGTLACKDLSIVGGSMGTTYVR
                                 |              |                        |                                    |       |          |
CBA120.TSP3-Ro103C3Iw.TSP     GYAGLYIEDYRDGSLMSSGTSNDIRLFQSRGYQHGVSMSRMQYTTMINCSVEECFKSPGETYAYAYKFIDPFSIHLSTCATEFVEGGQIQITGFANPGFR
                              ||||||||||||||||||||||||||||||||||||||||||||||||||||||||||||||||||||||||||||||||||||||||||||||||||||
Ro103C3lw.TSP                 GYAGLYIEDYRDGSLMSSGTSNDIRLFQSRGYQHGVSMSRMQYTTMINCSVEECFKSPGETYAYAYKFIDPFSIHLSTCATEFVEGGQIQITGFANPGFR

CBA120.TSP3                   GGYYTQPVGYSDISSNSTGILLSGVAFDEALTTKVYLDTSITTRNKVINCSGVPDTIARGSTANRPANPQASYQYYDTTLGIPIWWNSVSGTWKNAAGAD
                                         |     |                |       |        | |     |          |         |
CBA120.TSP3-Ro103C3Iw.TSP     PSITITEYFAVDQQNPATAHNIIDIDNGGVARCTVKVIGGDWTHDPRTPNVGSPRAAGSGTVVTTIGTTGAEGPNWLLSGGAKVVDIENATNTRSS
                              ||||||||||||||||||||||||||||||||||||||||||||||||||||||||||||||||||||||||||||||||||||||||||||||||
Ro103C3lw.TSP                 PSITITEYFAVDQQNPATAHNIIDIDNGGVARCTVKVIGGDWTHDPRTPNVGSPRAAGSGTVVTTIGTTGAEGPNWLLSGGAKVVDIENATNTRSS

CBA120.TSP3                   V

CBA120.TSP3-Ro103C3Iw.TSP

Ro103C3lw.TSP
```

D

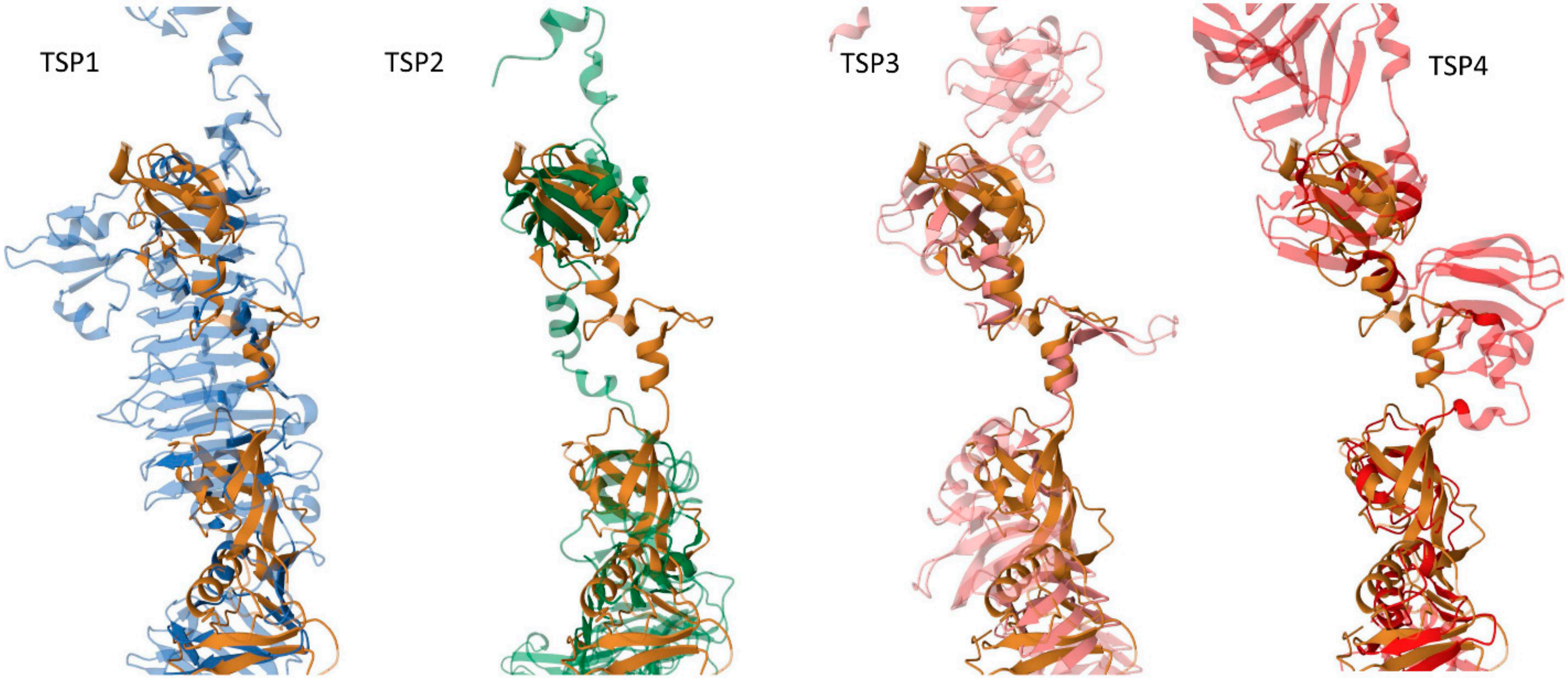

E

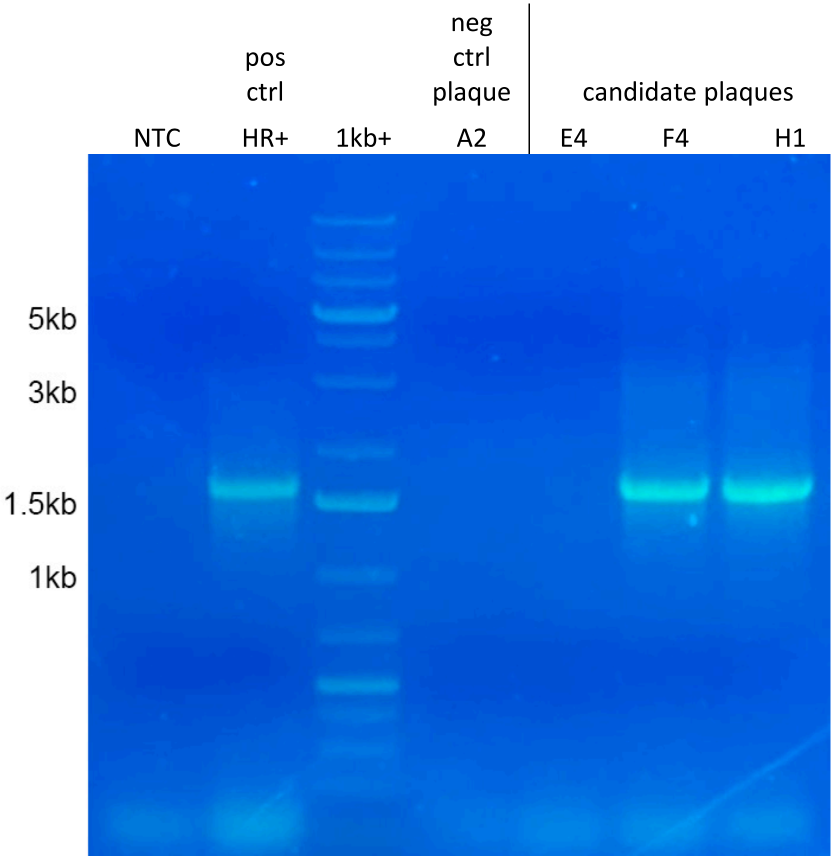

Figure S4: A) Homologous recombination cassette to generate O103 specific chimeric TSP3. B) TSP3 region of RBP-CBA120-6 with PCR primers to detect recombinant indicated. C) The amino acid sequences of the recipient, donor, and chimeric proteins were aligned based on splice site used with native CBA120 TSP3 shown in green and Ro103C3lw TSP in red. D) Pairwise Structure Alignment [5] of Alphafold2 predictions of Ro103C3lw (brown) TSP against CBA120 TSP1 (blue), TSP2 (green), TSP3 (light red) and TSP4 (dark red). Visual comparison shows highest overlap with TSP3. E) Agarose gel of PCR assay of 3 candidate recombinant plaques with two yielding bands at expected size (1585 bp), including negative control plaque (A2), positive PCR control (HR lysate), and no template control (NTC).

Supplementary Materials

Figure S5. RBP-CBA120-6 plaque Screen for Loss of O77 Tropism

A

O77							O45					
3.40E+04	5.70E+09	2.09E+09	2.68E+06	9.39E+08	5.62E+08		3.12E+04	3.32E+09	2.24E+09	8.59E+08	1.12E+09	7.69E+08
1.60E+09	1.17E+09	3.66E+09	2.04E+06	1.22E+09	4.50E+06		1.46E+09	1.12E+09	1.93E+09	6.58E+08	2.06E+09	1.03E+09
8.17E+06	1.09E+09	4.79E+06	2.26E+06	9.17E+08	4.43E+06		1.11E+09	1.44E+09	1.50E+09	8.69E+08	5.90E+08	1.48E+09
1.15E+09	1.83E+09	5.60E+08	3.90E+06	1.01E+06	8.29E+08		5.41E+08	2.81E+09	1.31E+09	5.27E+08	5.84E+08	9.75E+08
1.07E+09	5.32E+08	6.55E+06	4.69E+05	3.73E+08	3.71E+06		1.64E+09	5.65E+08	8.88E+08	5.87E+08	5.83E+08	6.10E+08
2.21E+09	8.27E+08	5.31E+06	1.66E+06	7.70E+08	4.25E+08		1.06E+09	6.70E+08	1.41E+09	1.38E+09	3.40E+08	6.98E+08
3.12E+06	7.18E+08	2.29E+09	4.97E+06	1.09E+09	6.28E+08		8.69E+08	8.31E+08	9.81E+08	1.12E+09	1.17E+09	8.63E+08
3.57E+06	1.27E+09	1.95E+04	6.16E+08	5.68E+06	1.22E+09		2.32E+09	2.31E+09	2.16E+04	8.85E+08	1.03E+09	9.93E+08

B

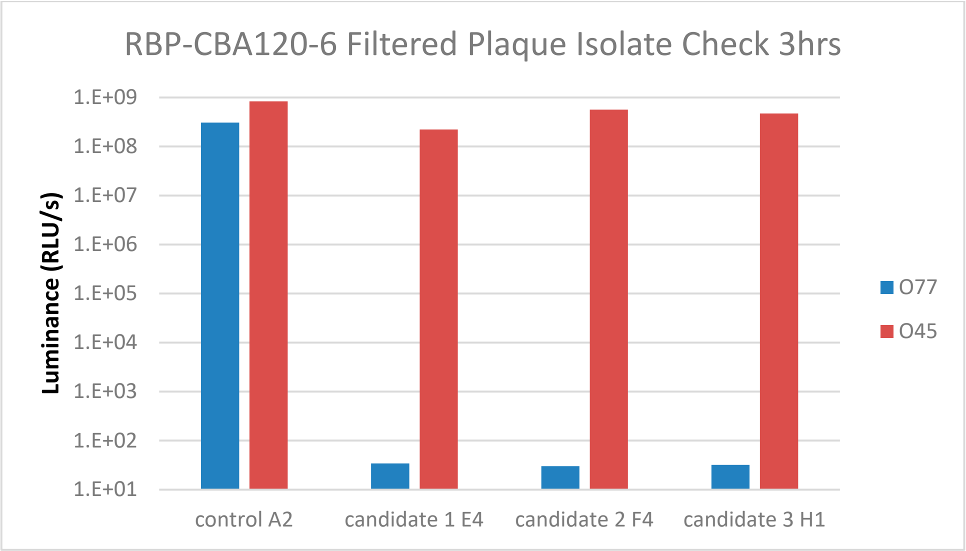

Figure S5: A) Parallel infections of plaque suspensions against *E. coli* O45 BAA-2193 and *E. coli* O77 ATCC 23537, heat map colors indicating high signal (red) and low signal (green), with candidate wells indicated in boxes based on luciferase activity ratios. B) Three-hour 37 °C infections were performed with the three candidate plaques along with the parental O77 positive control well (A2), verifying loss of O77 activity in candidate plaques.

Supplementary Materials

Figure S6. Luciferase Based Infection Assay to Detect O103 Activity

A

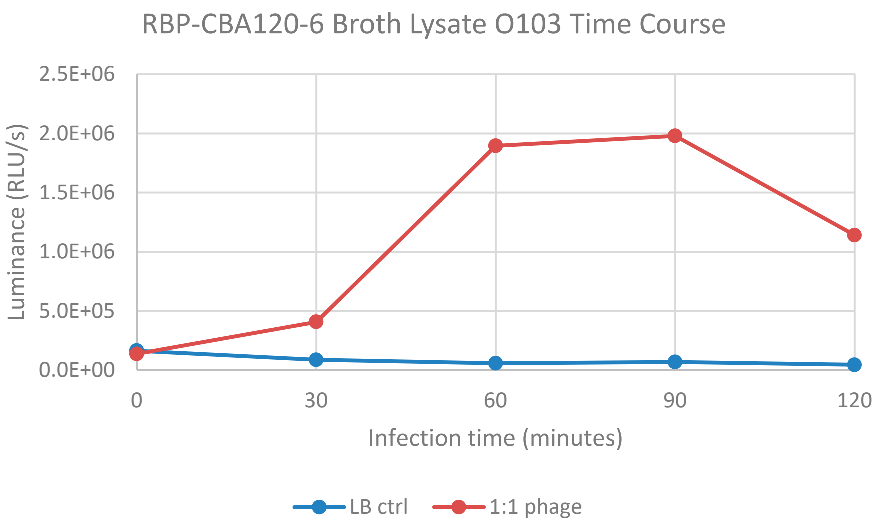

B

	0 minutes	30 minutes	60 minutes	90 minutes	120 minutes
LB control	164,128	87,532	58,176	69,060	45,321
RBP-CBA120-6	137,528	407,690	1,895,594	1,979,607	1,140,638
Signal/background	0.84	4.66	32.6	28.7	25.2

Figure S6: Time course of RBP-CBA120-6 broth lysate against *E. coli* O103 BAA-2210, demonstrating detection. A) Graph of luciferase data (RLU/s) from 5 µL samples of infection tested with 25 µL 1x NanoGlo® in Promega GloMax Navigator luminometer. B) Raw luciferase data given in RLU/s.

Supplementary Materials

Figure S7. RBP-CBA120-9 O26 Specific Chimera Cloning, Alignments and PCR

A

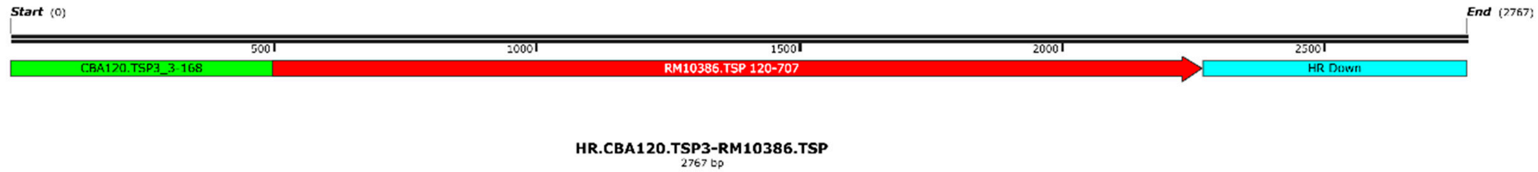

B

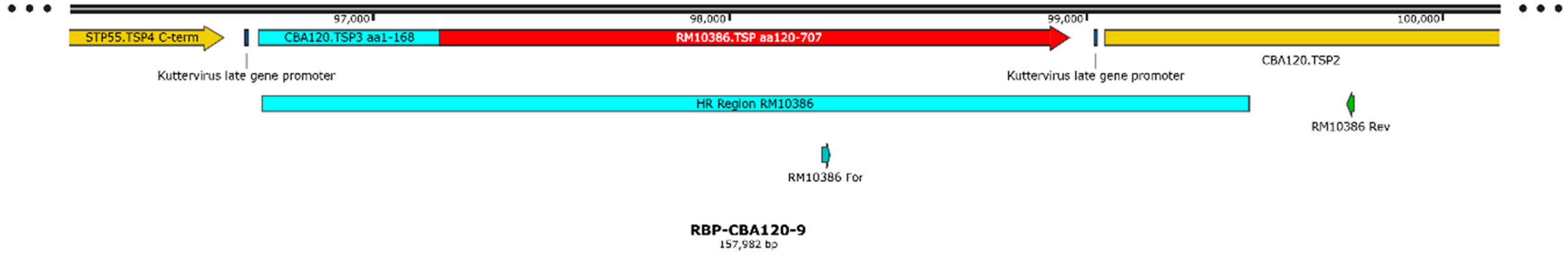

C

```
CBA120.TSP3               MISQFNQPRGSTSIEVNKQSIARNFGVKEDEVIYFTAGIDLSGFKVIYDESTQRAYSLPFGIVSGTTAISLDERAILTHSAGSVDLGELAVSREEYVTLP
                          ||||||||||||||||||||||||||||||||||||||||||||||||||||||||||||||||||||||||||||||||||||||||||||||||||||
CBA120.TSP3-RM10386.TSP   MISQFNQPRGSTSIEVNKQSIARNFGVKEDEVIYFTAGIDLSGFKVIYDESTQRAYSLPFGIVSGTTAISLDERAILTHSAGSVDLGELAVSREEYVTLP
                                                                                |           |
RM10386.TSP                                                                MTDITANVIVSMPSQLFTMARSFKAVANGKIYIGKIDTDPVNPENQIQVYV

CBA120.TSP3               GSFNFGHTINVKNELLVHDDKKYRWDGSLPKVVAAGSTPDSSGGVGLGAWLSVGDAALRAELNTKVSDGTFPATIKYKYGLPSVIDGAIYRTVQDKLDDF
                          ||||||||||||||||||||||||||||||||||||||||||||||||||||||||||||||||||||                           ||
CBA120.TSP3-RM10386.TSP   GSFNFGHTINVKNELLVHDDKKYRWDGSLPKVVAAGSTPDSSGGVGLGAWLSVGDAALRAELNTKVSDSEDHSLGDSLVMHNPQFVDSTSTILSKKLTID
                                |                        |   |         |                      ||||||||||||||||||||||||||||||||
RM10386.TSP               ENEDGSHVPVAQPIIINAGGYPVYNGQIAKFVTVQGHSMAVYDAYGAQQFYFPNVLKYDPDQFKGNLLSEDHSLGDSLVMHNPQFVDSTSTILSKKLTID
```

Supplementary Materials

```
CBA120.TSP3               VFLEDFGGKDDAGSTDNSIAFRKAFASGARKIRLRGSGVYGMATRDIELPAKYEIIGNAKNPEIKYLGTDTSFTMFTLTGSGPASNQWKQGGMFRDLIIS
                                 |           |        |                                                    |
CBA120.TSP3-RM10386.TSP   LRTLTDYGFAEGNTGAQNKAAFQKAIDDATLPTEIVIPEGAFIVDPGITIKNTVTMIRGAGAYQSRIFSTGTAAPIITQQDGVITFCEFRDFGLDGNGYA
                          ||||||||||||||||||||||||||||||||||||||||||||||||||||||||||||||||||||||||||||||||||||||||||||||||||||
RM10386.TSP               LRTLTDYGFAEGNTGAQNKAAFQKAIDDATLPTEIVIPEGAFIVDPGITIKNTVTMIRGAGAYQSRIFSTGTAAPIITQQDGVITFCEFRDFGLDGNGYA

CBA120.TSP3               SDVKINWMLGRHVQNLDYDRVFFYNSATVLNNYHYVNFTRCERWGSAFIGRADLNTIQFISESPKFHLCFSSGSPIDVWDTADLAITKCTMFAGDYAVRT
                                                  |   |          |        |          |  |
CBA120.TSP3-RM10386.TSP   ANGISLTEANHIKIENIDVVNTNNNAILVNGYSIDIIGCRLFQNAGNGINVGGYCNNINIINSRIYGNGAGGVLLTPAYAEGGMSVRVNGNNIEQNKFYG
                          ||||||||||||||||||||||||||||||||||||||||||||||||||||||||||||||||||||||||||||||||||||||||||||||||||||
RM10386.TSP               ANGISLTEANHIKIENIDVVNTNNNAILVNGYSIDIIGCRLFQNAGNGINVGGYCNNINIINSRIYGNGAGGVLLTPAYAEGGMSVRVNGNNIEQNKFYG

CBA120.TSP3               RVTQKQVTAPDLFAGYPVLITCSVFDAVRGHAWDLEGSVYSTITGNLVSAGRDTNSHGAYIKGGRSLSLTGNVFTYCGNYGLVLEDVQQSGFVGNVFNGN
                                                     |                  |       |
CBA120.TSP3-RM10386.TSP   LLAYGVKGLNLDANYWERNGEIGYPYSVPESITVRADIHLIANNFTLIPDLSKINDTVSIRGNQQTAIGYASALPNQDGFIFTNYAKNLTIENNQLLDAS
                          ||||||||||||||||||||||||||||||||||||||||||||||||||||||||||||||||||||||||||||||||||||||||||||||||||||
RM10386.TSP               LLAYGVKGLNLDANYWERNGEIGYPYSVPESITVRADIHLIANNFTLIPDLSKINDTVSIRGNQQTAIGYASALPNQDGFIFTNYAKNLTIENNQLLDAS

CBA120.TSP3               KTGGLGTLACKDLSIVGGSMGTTYVRGGYYTQPVGYSDISSNSTGILLSGVAFDEALTTKVYLDTSITTRNKVINCSGVPDTIARGSTANRPANPQASYQ
                          |   |       ||                   ||  |                  ||                       |
CBA120.TSP3-RM10386.TSP   KVNNLLAMYHNNLSSKVTDRLYLANNTVNSIGYVGSYDPATQNPDTAHLIDIANRELTANYLDRNMLLWTAASGTTGTLIKTQNIYAGNYSFLVTTGDRV
                          ||||||||||||||||||||||||||||||||||||||||||||||||||||||||||||||||||||||||||||||||||||||||||||||||||||
RM10386.TSP               KVNNLLAMYHNNLSSKVTDRLYLANNTVNSIGYVGSYDPATQNPDTAHLIDIANRELTANYLDRNMLLWTAASGTTGTLIKTQNIYAGNYSFLVTTGDRV

CBA120.TSP3               YYDTTLGIPIWWNSVSGTWKNAAGADV
                             |              |  |   |
CBA120.TSP3-RM10386.TSP   WGRTIDLNKSPELKGKFVWFGAWVNDQGSASKLMFIINGAGQTDSTAPLAGNGKWSYVSCGVYIYETDTAINVGIRNYGSGNVLINSPSLCAYGMPSNAL
                          ||||||||||||||||||||||||||||||||||||||||||||||||||||||||||||||||||||||||||||||||||||||||||||||||||||
RM10386.TSP               WGRTIDLNKSPELKGKFVWFGAWVNDQGSASKLMFIINGAGQTDSTAPLAGNGKWSYVSCGVYIYETDTAINVGIRNYGSGNVLINSPSLCAYGMPSNAL

CBA120.TSP3

CBA120.TSP3-RM10386.TSP   QVEKTTFYLSSVPTSGFWDIGERVINSAPASGQPKAWTCNIPGGPGTYSFLSEGNF
                          ||||||||||||||||||||||||||||||||||||||||||||||||||||||||
RM10386.TSP               QVEKTTFYLSSVPTSGFWDIGERVINSAPASGQPKAWTCNIPGGPGTYSFLSEGNF
```

D

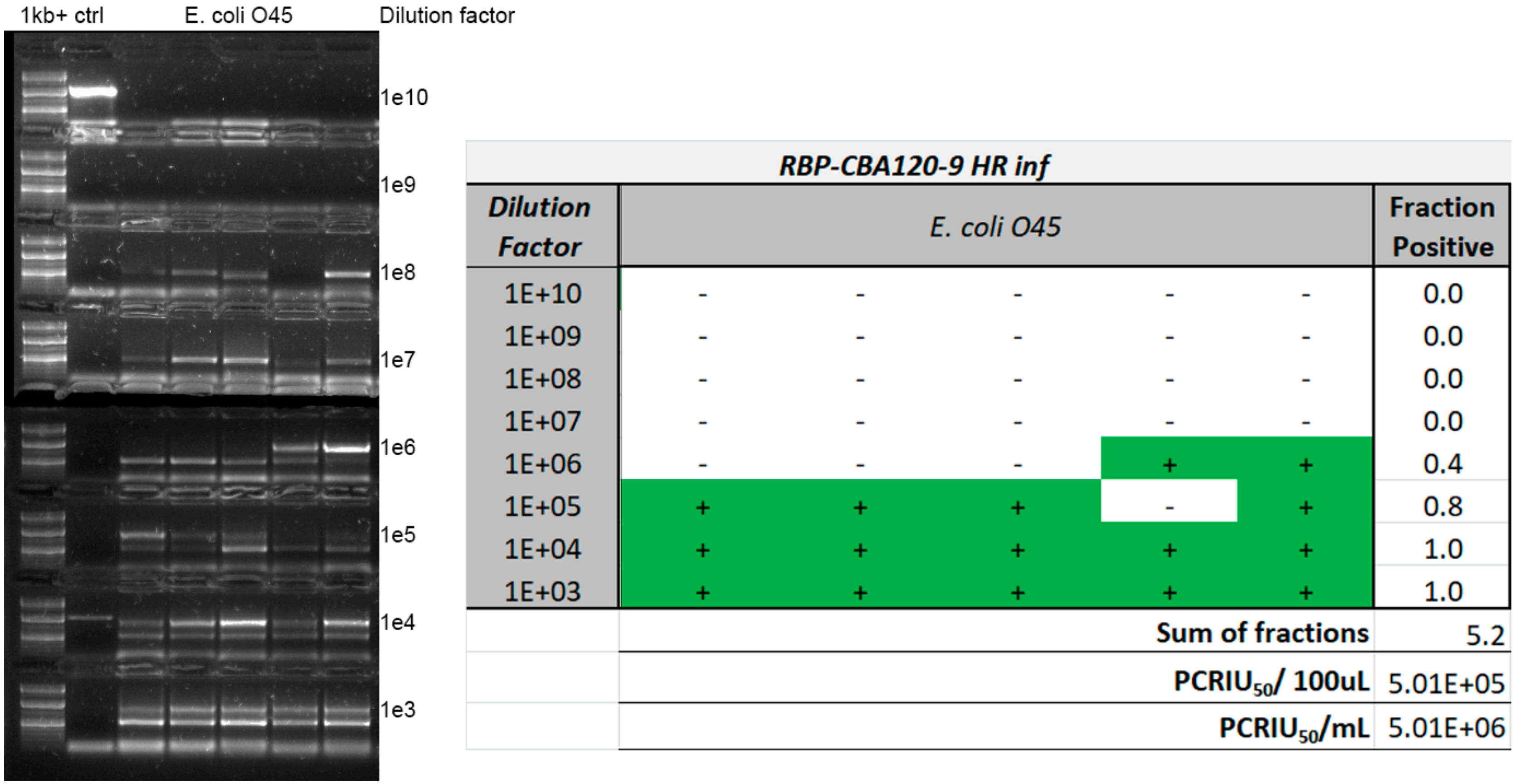

RBP-CBA120-9 HR inf						
Dilution Factor	*E. coli O45*					**Fraction Positive**
1E+10	-	-	-	-	-	0.0
1E+09	-	-	-	-	-	0.0
1E+08	-	-	-	-	-	0.0
1E+07	-	-	-	-	-	0.0
1E+06	-	-	-	+	+	0.4
1E+05	+	+	+	-	+	0.8
1E+04	+	+	+	+	+	1.0
1E+03	+	+	+	+	+	1.0
					**Sum of fractions**	5.2
					**$PCRIU_{50}$/ 100uL**	5.01E+05
					**$PCRIU_{50}$/mL**	5.01E+06

Figure S7: A) Homologous recombination cassette to generate O26 specific chimeric TSP3. B) TSP3 region of RBP-CBA120-9 with PCR primers to detect recombinant indicated. C) The amino acid sequences of the recipient, donor, and chimeric proteins were aligned based on splice site used, with native CBA120 TSP3 shown in green and RM10386 TSP in red. D) Agarose gel and scoring table of the PCRIU50 of the original homologous recombination infection lysate, with a positive control in the top well of the controls lane and no bacteria negative controls below. Limiting dilutions were scored as positive, and indicated on the adjacent chart, if the expected size band (1491 bp) was brighter than bands (if any) in the corresponding negative lane at the same dilution.

Supplementary Materials

Figure S8. Luciferase Based Infection Assay to Detect O26 Activity

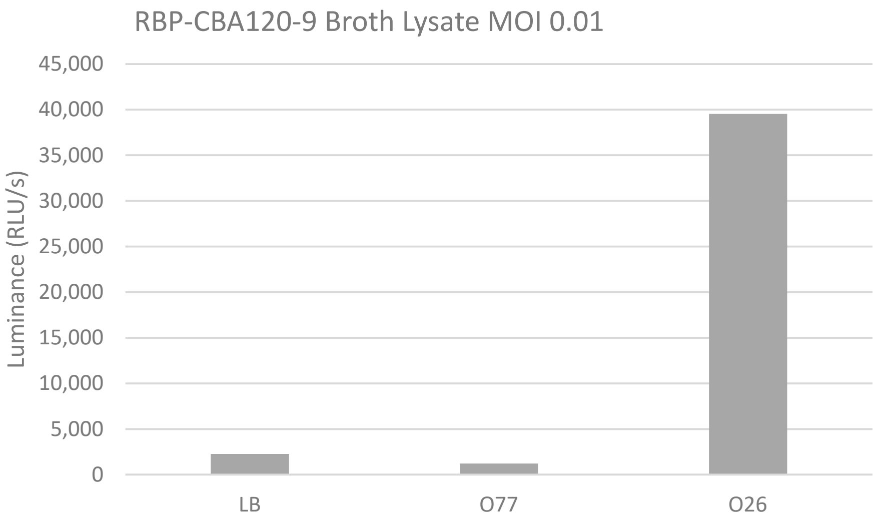

Figure S8: Two-hour infection of RBP-CBA120-9 broth lysate against *E. coli* O26 BAA-2205 and *E. coli* O77 ATCC 23537 in triplicate, demonstrating replacement of O77 specificity with O26. Graph of luciferase data (RLU/s) from 5 µL samples of infection tested with 25 µL of 1x NanoGlo® in Promega GloMax Navigator luminometer.

References

1. Gil, J.; Paulson, J.; Brown, M.; Zahn, H.; Nguyen, M.M.; Eisenberg, M.; Erickson, S. Tailoring the Host Range of Ackermannviridae Bacteriophages through Chimeric Tailspike Proteins. *Viruses* **2023**, *15*, 286, doi:10.3390/v15020286.
2. Rice, P.; Longden, I.; Bleasby, A. EMBOSS: The European Molecular Biology Open Software Suite. *Trends Genet.* **2000**, *16*, 276–277, doi:10.1016/S0168-9525(00)02024-2.
3. Needleman, S.B.; Wunsch, C.D. A General Method Applicable to the Search for Similarities in the Amino Acid Sequence of Two Proteins. *J. Mol. Biol.* **1970**, *48*, 443–453, doi:10.1016/0022-2836(70)90057-4.
4. Davis, M.W.; Jorgensen, E.M. ApE, A Plasmid Editor: A Freely Available DNA Manipulation and Visualization Program. *Front. Bioinforma.* **2022**, *2*, doi:10.3389/fbinf.2022.818619.
5. Berman, H.M.; Westbrook, J.; Feng, Z.; Gilliland, G.; Bhat, T.N.; Weissig, H.; Shindyalov, I.N.; Bourne, P.E. The Protein Data Bank. *Nucleic Acids Res.* **2000**, *28*, 235–242, doi:10.1093/nar/28.1.235.
6. Jumper, J.; Evans, R.; Pritzel, A.; Green, T.; Figurnov, M.; Ronneberger, O.; Tunyasuvunakool, K.; Bates, R.; Žídek, A.; Potapenko, A.; et al. Highly Accurate Protein Structure Prediction with AlphaFold. *Nature* **2021**, *596*, 583–589, doi:10.1038/s41586-021-03819-2.
